# Experimental medicine study with stabilised native-like HIV-1 Env immunogens drives long-term antibody responses, but lacks neutralising breadth

**DOI:** 10.1016/j.ebiom.2024.105544

**Published:** 2025-01-02

**Authors:** Katrina M. Pollock, Hannah M. Cheeseman, Leon R. McFarlane, Suzanne Day, Monica Tolazzi, Hannah L. Turner, Jennifer Joypooranachandran, Katsiaryna Shramko, Stefania Dispinseri, Philipp Mundsperger, Ilja Bontjer, Nana-Marie Lemm, Sofia Coelho, Maniola Tanaka, Tom Cole, Bette Korber, Dietmar Katinger, Quentin J. Sattentau, Andrew B. Ward, Gabriella Scarlatti, Rogier W. Sanders, Robin J. Shattock

**Affiliations:** a Imperial College London, Department of Infectious Disease, UK; b NIHR Imperial Clinical Research Facility and NIHR Imperial Biomedical Research Centre, London, UK; c Viral Evolution and Transmission Unit, Division of Immunology, Transplantation, and Infectious Diseases, IRCCS Ospedale San Raffaele, Milan, Italy; d Department of Integrative Structural and Computational Biology, The Scripps Research Institute, La Jolla, CA, USA; e Polymun Scientific Immunbiologische Forschung GmbH, Klosterneuburg, Austria; f Department of Medical Microbiology, Academic Medical Centre University of Amsterdam, Amsterdam, the Netherlands; g New Mexico Consortium, Los Alamos, NM, USA; h The Sir William Dunn School of Pathology, The University of Oxford, Oxford, UK

**Keywords:** HIV envelope, Experimental medicine, HIV-1, Prefusion, Trimer, Vaccine

## Abstract

**Background:**

We report findings from an experimental medicine study of rationally designed prefusion stabilised native-like HIV envelope glycoprotein (Env) immunogens, representative of global circulating strains, delivered by sequential intramuscular injection.

**Methods:**

Healthy adult volunteers were enrolled into one of five groups (A to E) each receiving a different schedule of one of two consensus Env immunogens (ConM SOSIP, ConS UFO, either unmodified or stabilised by chemical cross-linking, followed by a boost with two mosaic Env immunogens (Mos3.1 and Mos3.2). All immunogens were co-formulated with liposomal Monophosphoryl-Lipid A (MPLA) adjuvant, and volunteers were followed up for 28 days post final Mosaic booster injection. Participants gave written informed consent to join the study. The study is registered on ClinicalTrials.gov ID NCT03816137.

**Findings:**

Fifty-one participants (men n = 23 and women n = 28) aged 18–55 were enrolled. The seroconversion rate against Env was 100% with all participants having measurable anti-Env IgG antibodies after their second injection and throughout the study. Neutralisation was detected against the ConM pseudovirus in sera of those who had received both ConM and ConS immunogens. However, this activity was limited in breadth and was neither boosted nor broadened in those receiving the Mos3.1 and Mos3.2 immunogens. Neutralising antibody function correlated with binding to V1/V3 and V5 epitopes and peaked after the third injection.

**Interpretation:**

Rationally designed prefusion-stabilised native-like Env trimers are robustly immunogenic in a prime-boost schedule. When given alone they are insufficient to induce neutralising antibody titres of significant breadth, but they represent potentially valuable polishing immunogens after germline-targeting.

**Funding:**

European Aids Vaccine initiative (EAVI2020) received funding from EU Horizon 2020, grant number 681137. Structural studies were supported by the 10.13039/100000865Bill and Melinda Gates Foundation (INV-002916).

**Research in context**

**Evidence before this study**

We searched PubMed from database inception up to April 8, 2024, using the terms “Prefusion” AND “HIV-1” AND “trimer” AND “clinical trials.” One previous clinical trial assessed the immunogenicity of a single, structurally stabilised HIV-1 clade A envelope glycoprotein Env trimer (Trimer 4571; BG505 DS-SOSIP) in healthy HIV-negative adults.^1^ The goal of our experimental medicine trial was to evaluate the immunogenicity of prefusion stabilised trimers representative of global circulating strains formulated in a strong B cell adjuvant, in healthy HIV-negative adults.

**Added value of this study**

This experimental medicine study expands on the Trimer 4571 trial to use prefusion stabilised consensus-based HIV-1 Env trimers as immunogens to further interrogate the human B cell response to native-like trimers. Robust and sustained humoral responses were induced by prefusion stabilised consensus (ConM and ConS) immunogens with and without chemical cross-linking. Limited neutralisation was detected in sera of participants correlate with binding to V1/V3 and V5 epitopes on Env. However, these were not boosted in those receiving a further immunisation with mosaic immunogens.

**Implications of all the available evidence**

The results of this experimental medicine study of prefusion stabilised HIV-1 Env trimers informs ongoing vaccine efforts to define the human immune responses capable of producing broadly neutralising antibodies (bNAbs). Further investigation of the potential of prefusion stabilised Env immunogens to engage the human B cell repertoire will likely require iterative experimental studies that assess immune challenge with a wide range of structurally modified Env trimers.

## Introduction

Forty years on from the beginning of the HIV-1 pandemic the search for an effective preventative vaccine remains elusive. The trimeric HIV-1 envelope glycoprotein (Env) is the only virally-encoded surface glycoprotein, and is therefore the only viral antigen able to induce HIV-specific neutralising antibodies. It has substantial amino acid variation, immunodominant hypervariable regions, structural and conformational instability, an extensive glycan shield, and is expressed at low density on the surface of the HIV-1 viral particle – features that facilitate viral evasion of B cell responses during infection.^2^^,^^3^

Unlike SARS-CoV-2 infection, where the formation of neutralising antibody that can interact with circulating variants is common and occurs within ∼10 days from the onset of symptoms, broadly neutralising antibodies (bNAbs) against the far more diverse HIV-1 Env are inefficiently induced by natural infection. Nevertheless, ∼10–50% of people living with HIV-1 (PLWH) develop bNAbs against HIV-1, with ∼1% of individuals becoming elite neutralisers by developing bNAbs that target conserved epitopes on the Env trimer capable of neutralising multiple HIV-1 strains.^4^^,^^5^ These epitopes include the CD4-binding site (CD4bs), membrane proximal external region (MPER), V2 apex, certain glycans around the ‘V3 supersite’, silent face, gp120-gp41 interface and fusion peptide.^6^, ^7^, ^8^, ^9^ Most of these antibodies are unusual in structure with high levels of somatic hypermutation (SHM) and many have long complementarity-determining region heavy chain 3 (CDRH3) loops, required to penetrate the glycan shield.^10^ Furthermore, naturally occurring bNAbs often have poly- and auto-reactive properties, which could lead to negative selection of such B cell lineages when vaccinating seronegative individuals.^11^ During natural infection, bNAb induction has been associated with persistent high-level exposure to Env during uncontrolled HIV-1 replication.^12^ Throughout this time, rounds of SHM are thought to gradually select for maturing antibodies with enhanced breadth, potentially through iterative rounds of antibody driving resistance in Env, which in turn selects for antibodies that can accommodate the newly emergent resistance mutations.^13^ However, such sustained levels of Env exposure are challenging to replicate by vaccination and, while binding antibodies have been induced through experimental immunisation, rapid waning of the response is common and seroreversion is even an occasional feature of treated infection.^14^^,^^15^ To induce persistent bNAbs, these barriers could, in theory, be overcome with repeated exposure to immunogens designed to mimic the process occurring in infection. There is, however, limited understanding of how Env immunogens interact with the naive human B cell receptor (BCR) repertoire, and the cellular pathways required to induce bNAbs in seronegative individuals.^16^ Naïve, germline-encoded BCR repertoires of non-human species do not closely model the human repertoire.^17^, ^18^, ^19^ Thus, broad investigation of the potential of synthetic Env immunogens to engage the human B cell repertoire will likely require iterative experimental medicine studies that assess immune challenge with a wide range of structurally modified Env trimers.

The evident success of mRNA vaccines against SARS-CoV-2 was predicated on using pre-fusion proline-stabilised native-like trimers. Here, the introduction of proline residues at key positions maintained the trimers in the pre-fusion configuration preventing their disassociation into structures that are not conducive to the formation of neutralising antibodies.^20^ However, such strategies were derived from decades of iterative design, research and development leading to the manufacture of soluble prefusion stabilised native-like HIV-1 Env immunogens.^6^ Nevertheless, the extent to which such stabilised native-like immunogens can induce neutralising antibodies against HIV-1 in humans is not fully known.

Experimental medicine studies are investigations undertaken in humans relating to model systems to either identify mechanisms of pathophysiology or disease or demonstrate proof-of-concept evidence of the validity and importance of new discoveries or treatments.^21^ In this HIV-1 experimental medicine study, rationally-designed Env immunogens were produced as part of the European AIDS Vaccine Initiative (EAVI2020) and used as tools to interrogate the human B cell response to prefusion stabilised native-like trimers. These challenge immunogens incorporated design features to overcome some of the intrinsic viral immune evasion mechanisms.^22^ The model consensus gp140 Env trimers (consensus of consensus sequences of the major clades, a design intended to capture the repeated aspects among all common global strains in a native-like Env) were designed to focus B cell responses to epitopes common to all group M HIV-1 subtypes. Two design strategies were used to stabilise these in a native-like conformation and were termed ConM SOSIPv7 and ConS uncleaved pre-fusion optimised (UFO).^23^^,^^24^ The ConM SOSIP trimer included mutations that incorporate disulphide linkages between the gp120 and gp41 ectodomain to prevent their disassociation into monomer subunits and the I559P mutation to stabilise gp41 in its pre-fusion conformation. As an alternative strategy to present a native-like consensus immunogen, ConS UFO utilises a short flexible amino-acid linker to tether the gp120 and gp41 subunits together and insertion of a short linker sequence in gp41 to prevent helix formation. In addition, the ConS UFO contains the mutations A433C + I201C that stabilise the CD4 binding site and V570D that stabilises gp41. Together, these linkers and mutations reduce non-neutralising antibody binding while maintaining high quaternary and MPER-specific bNAb binding, with a rabbit immunisation study demonstrating the induction of autologous tier 2 neutralisation.^23^ To further stabilise the global architecture, 1-ethyl-3-(3-dimethylaminopropyl) carbodiimide hydrochloride (EDC) crosslinking was assessed for both immunogens. This has been shown to globally enhance immunogen biophysical stability, largely conserve bNAb epitopes, and reduce CD4 binding and off-target antibody responses.^25^^,^^26^

As a second component to the study, two mosaic gp140 Env trimers (Mos3.1 and Mos3.2) were manufactured to explore their potential as boosting immunogens to further reduce unwanted immunodominant type-specific antibody responses and guide B cells towards highly conserved supersites of vulnerability on Env, with particular emphasis on quaternary bNAb epitopes.^27^, ^28^, ^29^ These mosaic immunogens were previously designed using computer algorithms to focus antibody responses towards globally conserved neutralisation epitopes. Like the ConM SOSIP trimer described above, we applied a SOSIP design strategy to preserve their pre-fusion native-like structure when expressed as soluble immunogens.

In this experimental medicine study, we sequentially primed the immune response with one of the consensus immunogens (ConM or ConS, with or without EDC cross linkage). The response was then rechallenged with the cocktail of two mosaic immunogens (Mos3.1 and Mos3.2). All immunogens were given as an injection with a liposomal adjuvant preparation of Monophosphoryl-Lipid A (MPLA) to enhance the response. Outcomes were assessed by measuring antigen-specific IgG antibody titres and the HIV-1 viral neutralisation activity of the induced serum antibodies as well as the characterisation of blood B and T cell responses. Electron microscopy-based polyclonal epitope mapping (EMPEM) was used to visualise the immunodominant serum antibody responses and determine their epitopes. Whilst a durable binding antibody response was elicited, neutralisation function was only raised against the autologous virus.

## Methods

### Ethics statement

The study was sponsored by Imperial College London (ICL). Approval for EAVI2020_01 was granted by the London–Fulham Research Ethics Committee (Ref:18/LO/2096; IRAS 251930). The study was conducted according to the principles of the Declaration of Helsinki (2008) and complied with the International Conference on Harmonization Good Clinical Practice guidelines adopted by the Sponsor for experimental medicine research. Written informed consent was obtained from all participants.

### Study design

The EAVI2020_01 (NCT03816137) study was a single-blinded three-part experimental medicine study. Experimental medicine refers to investigations undertaken in humans relating to model systems to either identify mechanisms of pathophysiology or disease or demonstrate proof-of-concept evidence of the validity and importance of new discoveries or treatments.^21^

We report data obtained from the first part of the study (Groups A to E).

The clinical phase of the study was conducted at the NIHR Imperial CRF, Hammersmith Hospital, Imperial College Healthcare NHS Trust, London, UK, between March 2019 and August 2022 (clinicaltrials.gov: NCT03816137). Fifty-one participants were recruited into the study (Supplementary Table S1). Participants were eligible if they were aged 18–55 years, had a negative HIV-1 test result at screening, fully comprehended the purpose and details of this study as provided in the Participant Information Sheet and were able to provide written informed consent. Eligibility assessment was by the results of laboratory tests, review of medical history, and, where appropriate, physical exam results. Participants with medical, psychological or other conditions, clinically significant laboratory result at screening, or use of any medications which, in the opinion of the investigators, would interfere with the study objectives or volunteers’ safety were ineligible. A medical event on a similar study using similar immunogens and the MPLA adjuvant (NCT03961438), prompted the study team to alter the exclusion criteria during the enrolment period, with the addition of history of angioedema and history of urticaria deemed significant by the Chief Investigator (see Supplementary Table S2 for full eligibility criteria). Additional volunteers could be enrolled to replace early withdrawals. To ensure the wellbeing of the participants and ethical conduct of the study, observations of adverse events including injection site and systemic reactogenicity were reviewed by the Data Monitoring Safety Committee (DMSC) at planned intervals during the study.

### Randomisation and masking

**Fig. 1:**
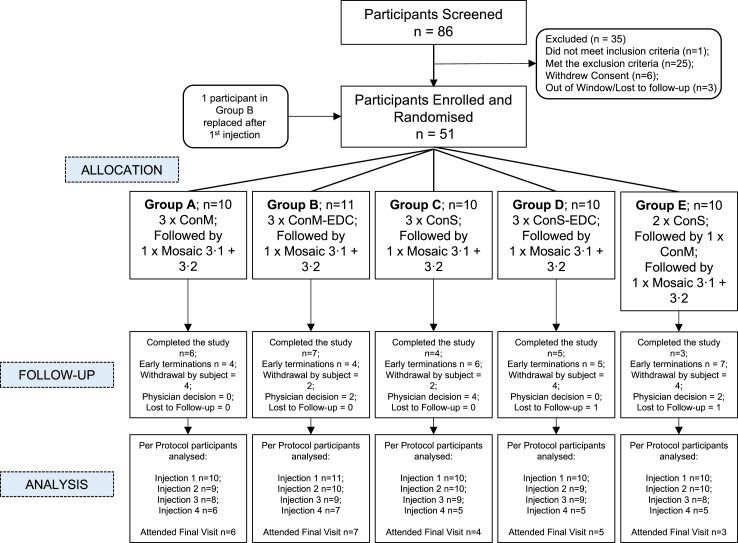
**CONSORT diagram of EAVI_01.** Diagram showing the flow of the experimental medicine study from screening through to completion of the final visits.

The study employed a single-blind with volunteers block-randomised into five groups of n = 10 (Groups A-E, Fig. 1) with the assignment of participants revealed at enrolment of the participant to the clinical study team (clinical research nurse and clinical research fellow) by means of sharing the next item on a concealed randomisation list. The random allocation sequence was generated by the sponsor’s Clinical Project Manager, using the List Randomiser service at RANDOM.ORG. All volunteers received Env immunogens but were masked to the regimes. The clinical research team were not masked to study allocation. Laboratory teams undertaking immunological analysis were masked to group and dosing regimen to prevent analysis bias. The clinical team delivering the study were not masked to Env immunogen schedule allocation.

### Safety monitoring during the experimental medicine study

Adverse event data were collected to monitor the safety of the participants for reporting to the Data Monitoring Safety Committee (DMSC) to ensure the study’s ethical conduct. There were no safety objectives of the study, which was not a clinical trial of an investigational medicinal product (CTIMP). Three safety reports detailing the reported systemic and localised adverse events, relationship to injection and expectedness assessment were submitted to the DMSC during the course of the study. The first DMSC report was triggered by a pause to the study after a grade 3 injection site and systemic reaction, which resolved fully. Grade 1–3 systemic or localised AEs considered possibly, probably or definitely related to the injections were included in the first study report and its supplement. The DMSC considered the overall level of local and systemic ARs and duration were as would be expected for adjuvanted vaccines and the overall profile of local and systemic ARs was in keeping with expected response to MPLA. The DMSC recommended that the Study Team continue with the current protocol with no alteration of dose levels of MPLA or immunogens, schedule or number of immunisations.

The DMSC recommended a structured questionnaire for reports two and three and diary card for recording adverse reactions be introduced after the first report. The structured questionnaire and diary card included grade 1–3 erythema/redness, pain, swelling at the injection site and feverishness, raised temperature, chills/shivers, myalgia, arthralgia, fatigue, headache, nausea, vomiting. Unsolicited adverse events were also considered. There were no safety concerns or further changes arising from the second or third reports. Development of the study injections for use as a vaccine against HIV would require additional structured safety assessment in a CTIMP, with the associated Development Safety Update Reports (DSURs).

### Production of immunogens

The ConM and ConS truncated soluble native-like gp140 Env glycoprotein trimers were developed and characterised as previously described.^23^^,^^24^ ConM is based upon the sequence of all group M isolates and stabilised by an SOS disulphide bond, whereas ConS is a SOSIP—UFO combined design of a group M consensus soluble trimer. GMP manufacture and affinity purification of native-like trimers was performed by Polymun (Austria) as described,^30^ and the biophysical and antigenic characterisation of the GMP trimers with and without EDC crosslinking has been previously reported.^30^

### Procedures

Model immunogens based on HIV-1 Env were given as intramuscular injections in 0.6 mL; ConM SOSIP, EDC ConM SOSIP, ConS UFO, EDC ConS UFO, Mos3.1 and Mos3.2 as one of five different schedules (Groups A to E). All schedules included a prime and two booster injections with a ConM SOSIP and/or ConS UFO (with and without EDC stabilisation) at 0, 3 and 6 months (Part 1). The final injection of Mos3.1 and Mos 3.2 was given to all groups at 12 months (Part 2). The different prime-boost combinations are summarised (Table 1). The ConM SOSIP and ConS UFO were given at a dose level of 100 μg and the Mosaic immunogens at 2 × 50 μg each (prepared as a single injection). All injections included liposomal MPLA at a dose level of 500 μg. All immunogens were administered into the deltoid muscle of the arm.

**Table 1:** Summary of immunogens and dosing schedule.

Group	N	Months			
		0	3	6	12
		Part 1			Part 2
A	10	ConM SOSIP 100 μg	ConM SOSIP 100 μg	ConM SOSIP 100 μg	Mos3.1 + Mos3.2 (2 × 50 μg)
B	10	EDC ConM SOSIP 100 μg	EDC ConM SOSIP 100 μg	EDC ConM SOSIP 100 μg	Mos3.1 + Mos3.2 (2 × 50 μg)
C	10	ConS UFO 100 μg	ConS UFO 100 μg	ConS UFO 100 μg	Mos3.1 + Mos3.2 (2 × 50 μg)
D	10	EDC ConS UFO 100 μg	EDC ConS UFO 100 μg	EDC ConS UFO 100 μg	Mos3.1 + Mos3.2 (2 × 50 μg)
E	10	ConS UFO 100 μg	ConS UFO 100 μg	ConM SOSIP 100 μg	Mos3.1 + Mos3.2 (2 × 50 μg)

### PBMC separation

Peripheral blood mononuclear cells (PBMC) were isolated using density gradient separation from heparinised whole blood. Cells were processed within 4 h of collection and stored at 1 × 10^7^ cells/mL in freezing media (10% dimethyl sulfoxide, 90% foetal bovine serum; Sigma–Aldrich) at −80 °C before storage in ultra-low −150 °C conditions prior to analysis.

### Detection of antigen-specific IgG antibodies

Antigen-specific IgG antibodies were measured in heat-inactivated sera using a standardised ELISA platform. In brief, 96-well high-binding plates (Greiner, Kremsmünster, Austria) were coated with anti-human kappa and lambda light chain specific mouse antibodies (Southern Biotech, Birmingham, AL) at 1:1 ratio diluted 1:500 in PBS or 1 μg/mL of the EAVI2020_01 proteins (ConM, ConS, ConM-EDC, ConS-EDC, Mosaic 3.1 or Mosaic 3.2) for 1 h at 37 °C. After blocking with block buffer (5% BSA (Sigma–Aldrich, St. Louis, MO), 0.05% Tween 20 (Fisher, Pittsburgh, PA) in D-PBS (Sigma–Aldrich) samples were initially screened in triplicate at 1:100 dilution, then further titrated, where needed, to obtain optimal dilutions. Serial dilutions (1:5) of immunoglobulin standards (purified human IgG starting at 1 μg/mL, respectively) were added in triplicate to kappa/lambda capture antibody-coated wells and incubated for 1 h at 37 °C. Secondary antibody, HRP-conjugated anti-human IgG (Sigma–Aldrich, St. Louis, MO), was added at 1:20,000 dilution and incubated for 1 h at 37 °C. Plates were developed with SureBlue TMB substrate (KPL, Insight Biotechnology, London, UK). The reaction was stopped after 5 min by adding TMB stop solution (KPL, Insight Biotechnology) and the absorbance read at 450 nm on a VersaMax 96-well microplate reader (Molecular Devices, Sunnyvale, CA). Data were analysed using SoftMaxPro Version 7.

### Neutralisation assay

Env-pseudotyped viruses ConM and ConS were produced in HEK293T cells [HEK293T (RRID:CVCL_0063)], titered on HeLa TZM-bl cells [TZM-bl (RRID:CVCL_B478)], and used in the TZM-bl assay to determine neutralising antibody responses.^31^ All cells lines were mycoplasma tested upon arrival and every 6 months. Duplicates of six steps of threefold dilution, starting with 1:20 of each serum obtained at visit 1, 14, 17 and 20, were incubated with viral supernatant (at relative luminescence units [RLU] between 148,000 and 272,000) for 1 h. Thereafter, 10^4^ TZM-bl cells were added, and plates were incubated for 48 h, when luciferase activity was measured by addition of Bright-Glo Luciferase assay system (Promega, Madison, WI). Neutralisation titres were defined as the sample dilution at which RLU were reduced by 50% compared to virus control wells after subtraction of background RLU in control wells with only cells and were calculated with a linear interpolation method using the mean of the duplicate responses.

### Electron microscopy-based polyclonal epitope mapping (EMPEM)

EMPEM^32^ was conducted following the protocol described in Turner et al.^33^ CaptureSelect IgG-Fc (multispecies) resin was added to 1 mL of human sera. IgG was digested on resin for 5 h using 1% w/w papain. 1 mg of polyclonal Fab was added to 15 μg of Env trimer and incubated overnight at 4 °C. Following size exclusion chromatography (SEC), the peak corresponding to the Env–Fab complex was concentrated to roughly 0.04 mg/mL and immediately added to negative stain EM (nsEM) grids. The grids were screened for stain quality and concentration then saved for data collection at a later day. nsEM grids were loaded onto a Tecnai F200 electron microscope equipped with a Tietz F416 camera and collection was done using Leginon.^34^ Micrographs were processed in Appion^35^ and particles were picked using DoG Picker^36^ followed by MSA/MRA^37^ stacking. Stacked particles were put into Relion^38^ and processed into 2D classes. 3D classification was performed on particles in 2D classes that resembled Env complexes. 3D maps containing Env–Fab complexes were isolated and refined. Using UCSF Chimera,^39^ Fab volumes were isolated and overlayed with an unliganded Env trimer to represent all epitopes that were seen in the serum sample.

### Intracellular cytokine staining

T cell responses were evaluated by intracellular cytokine staining (ICS) for CD8^+^ and CD4^+^ antigen-specific responses. All antibodies were pre-titrated to optimal dilutions. ICS was performed on PBMC isolated pre-injection (visit 1), 2 weeks post-third injection (visit 14), at the time of the Mosaic Boost (visit 17) and 2 weeks post-mosaic boost (visit 20). Cryopreserved PBMC were thawed and rested overnight in R10 media at 37 °C, 5% CO_2_. After overnight incubation, 1 × 10^6^ viable PBMC were stimulated for 6 h at 37 °C with 1 μg/mL of either the Consensus 1, Consensus 2, Mosaic 1 or Mosaic 2 Peptide Pools (15-mers, overlapping by 11) matching the immunogen constructs. Cell stimulation cocktail (Phorbol 12-myristate 13-acetate (PMA)/Ionomycin (eBioscience, San Diego, CA) diluted 1:500) and CEFX Ultra SuperStim pool (JPT Peptide, Berlin, Germany) 1 μg/mL) were used as positive controls and R10 media with matched DMSO concentration used as a negative control. Two hours into the stimulation, brefeldin A (eBioscience, San Diego, CA) was added. After antigen stimulation, cells were stained with fixable viability dye eFluor780 (eBioscience, San Diego, CA), CD3 BV650 (Clone OKT3), CD4 PerCP-Cy5.5 (RPA-T4), CD8 AF700 (RPA-T8), IFN-γ AF488 (4S.B3), TNF-α PE-Cy7 (MAb11), IL-2 BV510 (MQ1-17H12), CD154 BV421 (24–31), (BioLegend, San Diego, CA) to assess CD4/CD8 specific responses. Cells were stained with fixable viability dye eFluor780 at room temperature for 30 min, followed by washing and staining with 100 μL/well surface markers (CD3, CD4, CD8 in 2% human serum) for 20 min. Cells were washed and fixed with 100 μL/well intracellular (IC) fixation buffer (eBioscience, San Diego, CA) at room temperature for 20 min, then washed twice with intracellular (IC) Permeabilisation buffer (eBioscience, San Diego, CA). Intracellular stain (IFN-γ, TNF-α, IL-2 and CD154) in assay buffer (PBS with 5% HI-FCS and 0.05% sodium azide) was added in a total of 100 μL/well at room temperature for 20 min, followed by 2 washes with IC Permeabilisation buffer. The cells were resuspended in Fixation buffer (eBioscience, San Diego, CA) and stored at 2–8 °C for no longer than 18 h prior to flow cytometry analysis on a Becton Dickinson Fortessa LSRII equipped with 50 mW 405 nm, 50 mW 488 nm, 50 mW 561 nm, 20 mW 633 nm lasers and a ND1.0 filter in front of the FSC photodiode. Acquisition was set to record 50,000 live CD3^+^ lymphocytes after dead cell and doublet exclusion (FSC-A/W, SSC-A/W gating). Analysis was performed using FlowJo software (Treestar, Ashland, OR). Data was expressed as percentage of total live CD4^+^ or CD8^+^ cells. Background responses in negative controls were subtracted from the stimulated samples.

### Activation induced marker assay

The Activation Induced Marker (AIM) assay was performed on PBMC isolated on pre-injection (visit 1), 1 week post-third injection (visit 13), at the time of the Mosaic boost (visit 17) and 2 weeks post-Mosaic boost (visit 20). All antibodies were pre-titrated to optimal dilutions.

Cryopreserved PBMC were thawed and rested at 5 × 10^6^ cells/mL for 3 h in 10% human assay buffer (10% HAB; 10% human serum (Sigma–Aldrich, St. Louis, MO) in RPMI media) at 37 °C, 5% CO_2_. After resting, 0.5 μg/mL CD40 blocking antibody (Miltenyi Biotech, Bergisch Gladbach, Germany) and CXCR5-BB515 (Clone RF8B2; BD Biosciences, San Diego, CA) was added for a 15 min incubation at 37 °C. Cells were stimulated for 18 h at 37 °C with media only, 1 μg/mL of either the Consensus 1, Consensus 2, Mosaic 1 or Mosaic 2 Peptide Pools (15-mers, overlapping by 11) matching the immunogen constructs. SEB (Staphylococcal enterotoxin B; Sigma–Aldrich, St. Louis, MO; at 1 μg/mL) and CEFX Ultra SuperStim pool (JPT Peptide, Berlin, Germany) were used as positive controls. After antigen stimulation, cells were stained with fixable viability dye eFluor506 (eBioscience, San Diego, CA), CD3 BUV395 (UCHT1), CD4 BUV496 (SK3), CD8 V500 (RPA-T8), CD14 V500 (M5E2), CD19 V500 (H1B19), CD154 PE (TRAP-1; all BD Biosciences, San Diego, CA), CD45RA PE-Dazzle (HI100), PD-L1 PE-Cy7 (29E.2AE), OX40 APC (ACT35), CD25 APC-Fire750 (BC96), CD69 BV650 (FN50) and PD-1 BV421 (EH12.2H7; all BioLegend, San Diego, CA). Cells were resuspended in Fixation buffer (eBioscience) and stored at 2–8 °C for no longer than 18 h prior to flow cytometry analysis measured on a Becton Dickinson FortessaLSR-SORP equipped with 20 mW 355 nm, 50 mW 405 nm, 50 mW 488 nm, 50 mW 561 nm, 20 mW 633 nm lasers and a ND1.0 filter in front of the FSC photodiode. Acquisition was set to record 50,000 live CD3^+^ lymphocytes after dead cell and doublet exclusion (FSC-A/W, SSC-A/W gating). Analysis was performed using FlowJo software (Treestar, Ashland, OR). Data was reported as % of parent population.

### IFN-γ ELISpot assay

T Cell IFN-γ ELISpot was performed on PBMC isolated on pre-injection (visit 1), 2 weeks post-third injection (visit 14), time of Mosaic boost (visit 17) and 2 weeks post-Mosaic boost (visit 20). Cryopreserved PBMC were thawed and rested overnight in R20 media (RPMI 1640 Media supplemented with 20% HI-FCS) at 37 °C, 5% CO_2_. Pre-coated IFN-γ plates were washed with 1xPBS and blocked with R10 for at least 1 h. After overnight incubation, 4 × 10^6^ viable PBMC were stimulated for 18–24 h at 37 °C, 5% CO_2_ with 1 μg/mL of either the Consensus 1, Consensus 2, Mosaic 1 or Mosaic 2 Peptide Pools (15-mers, overlapping by 11) matching the immunogen constructs. R10 alone was used as the negative control and 1 μg/mL CEFX Ultra SuperStim pool and 2.5 μg/mL α-CD3 (α-CD3 wells contained 4 × 10^5^ cells/well) were used as positive controls. After incubation, plates were washed and incubated for 2 h at room temperature with primary antibody, 1 μg/mL anti-human IFN-γ biotin. After washing, Streptavidin ALP solution was added and incubated for 1 h at room temperature, then developed with BCIP/NBT substrate for approximately 5–7 min before stopping the reaction with water. Plates were stored overnight in the dark to dry before being read with an automated AID iSpot ELISpot reader (Autoimmun Diagnostika GmbH, Straβberg, Germany). The number of spot-forming units per million cells or SFU/M, was calculated as the mean count minus the background count.

### Memory B cell ELISpot assay

B-cell enzyme-linked immunospot (ELISpot) was used to assess the magnitude of antigen-specific memory B cell (mBC) responses. Cryopreserved PBMC isolated on pre-injection (visit 1), 4 weeks post-third injection (visit 15), day of mosaic boost (visit 17) and 4 weeks post-mosaic boost (visit 21) were thawed and resuspended at 1 × 10^6^ cells/mL in stimulation media composed of 5 ng/mL recombinant human interleukin-2 (IL-2) (Roche, Welwyn Garden City, United Kingdom) and 0.5 μg/mL R848 (InvivoGen, San Diego, CA) for 4 days at 37 °C, 5% CO_2_. Sterile 96-well ELISpot plates (Millipore, Billerica, MA) were pre-wet with 15 μL/well 70% ethanol for 1 min before washing with sterile water. Wells were coated overnight at 2–8 °C with 5 μg/mL of the EAVI2020_01 proteins (ConM, ConS, ConM-EDC, ConS-EDC, Mosaic 3.1 or Mosaic 3.2), 15 μg/mL of anti-human IgG capture antibody MT91/145 (Mabtech, Stockholm, Sweden) or PBS. The following day, plates were blocked with RPMI supplemented with 10% heat-inactivated foetal calf serum (HI-FCS; R10) for 1–2 h before the addition of 10,000 cells/well for total IgG and 200,000 cells/well for antigen-specific responses. Each condition was tested in triplicate and plates were incubated for 6 h at 37 °C, 5% CO_2_. Biotinylated detection antibody MT78/145 (Mabtech) was added at 1 μg/mL in PBS/0.5% FCS and incubated overnight at 2–8 °C. The next day, streptavidin-HRP (Mabtech) diluted 1:1000 in PBS/0.5% FCS was added and incubated for 1 h at room temperature. AEC substrate solution (BD Biosciences, San Diego, CA) was added and incubated for 5–10 min before stopping the spot development with tap water. Plates were left to dry overnight, and spot-forming units (SFU) were counted using an automated AID iSpot ELISpot reader (Autoimmun Diagnostika GmbH, Straβberg, Germany). Data was reported as spot-forming units per million cells (SFU/M) and was calculated as the mean count minus the background count. The % antigen positive was calculated as a % of the total IgG positive events.

### Sample size

An estimate of the power of group comparisons using quantitative antibody titre was dependent on the number of responders. The sample size was based on the previous studies of HIV-1 gp140 immunogens.^40^, ^41^, ^42^, ^43^ By the end of this study, 8–10 volunteers were exposed to each schedule and this provided confidence around the response/event proportions of 0–100% in Table 2.

**Table 2:** Sample size.

^1,2^Clopper-Pearson or Wilson method (suitable for small sample sizes).

### Statistical analyses

An intention to treat (ITT) analysis has been performed throughout.

The primary outcome was the measurement of specific viral neutralisation activity of serum antibodies. Exploratory outcomes included titres of binding antibodies in serum and the characterisation of blood B and T cell responses. To assess the difference between groups at given timepoints, a Kruskal–Wallis Test with Dunn’s multiple correction test was performed in all assays. In addition, a Spearman’s correlation was performed to asses the correlation between the viral neutralisation activity and titres of serum binding antibodies to ConM and ConS. All statistical analyses were performed using GraphPad Prism version 10.0.2.

### Role of funders

The funders had no role in the study design, the collection, analysis and interpretation of data, writing of the report and the decision to submit for publication.

## Results

### Participants

Fifty-one participants (men n = 23 and women n = 28) aged 18–65 were enrolled. This included one participant in group B who was withdrawn, and an additional participant replaced as per the protocol. The total number of participants to continue in the study was n = 50. The volunteer recruitment and retention are detailed in the CONSORT diagram (Fig. 1). The majority (43/50) of participants completed Part 1 of the study, receiving the first three injections of a ConM SOSIP and/or ConS UFO Env immunogen (Table 1). An additional Group assessed the impact of priming with ConM (two injections) followed by boosting with ConS to determine any impact on neutralisation breadth. The study was interrupted by the COVID-19 pandemic, which enforced a pause due to prioritisation of COVID-19 research. Following lifting of the pause, 24/50 (48.0%) participants returned to complete Part 2 (injection of Mos3.1 and Mos 3.2, Table 1). In line with the approved study objectives as this was an experimental medicine, this study did not formerly assess safety parameters of the study injections. To maintain the safety of the participants, safety was continuously monitored by the study team and at planned intervals by the Data and Safety Monitoring Committee (DSMC). There was a requirement to report unexpected serious adverse events considered related to the study injection to the Sponsor and the research ethics committee who approved the study, and no such events occurred.

### Robust and durable serological anti-Env response

**Fig. 2:**
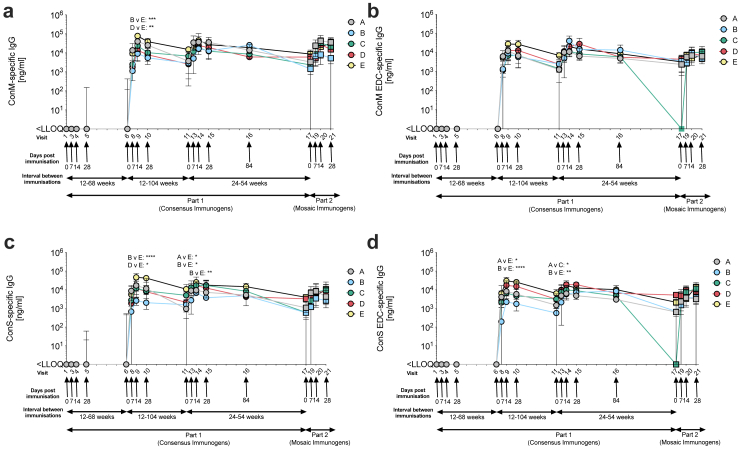
**Antigen-specific IgG responses in serum samples from participants of the EAVI2020_01 experimental medicine study.** The concentration of IgG specific for **(a)** ConM, **(b)** ConM-EDC, **(c)** ConS or **(d)** ConS-EDC were assessed. In part 1 (circles) Group A (Grey) received three injections with 100 μg ConM, Group B (Light Blue) received three injections with 100 μg ConM-EDC, Group C (Green) received three injections with 100 μg ConS, Group D (Pink) received three injections with 100 μg ConS-EDC and Group E (Yellow) received two injections with 100 μg ConS followed by one injection with 100 μg ConM. Injections 1–3 were given at visits 1, 6 and 11. All groups were boosted with a cocktail of 50 μg Mosaic 3.1 and 50 μg Mosaic 3.2 during the fourth injection (visit 17; part 2 (squares)). All injections were adjuvanted with 500 μg MPLA. The arrows indicate the timepoints after injection at which blood was taken for immunogenicity analysis. Median values with IQR are shown. The Kruskal–Wallis with Dunn’s multiple correction test was performed to compare the statistical differences between groups at day 0, 7, 14, 28 and 84 post injection. ∗ <0.05; ∗∗<0.01; ∗∗∗ <0.001; ∗∗∗∗ <0.0001.

Serum from participants in all groups was tested for IgG antibodies against ConM, ConS, ConM-EDC and ConS-EDC prior to and after receiving each injection (Fig. 2). IgG anti-Env antibodies were induced in sera from all participants who receive more than one injection (n = 50), with 100% seroconverting to Env after the second injection (Fig. 2). In each group receiving only ConM or ConS ( ± EDC), the highest IgG titre was against the matched immunogen, e.g., participants in group A receiving the ConM immunogen, induced the highest anti-ConM IgG concentrations compared with other groups receiving a different schedule of immunogens (Table 2). Group E receiving (ConS-ConS-ConM) induced potent responses against all immunogens. Antibody responses to all antigens (ConM, ConS and the EDC variants) were detected up to 104 weeks post-second injection in all groups (Fig. 2).

### 1-ethyl-3-(3-dimethylaminopropyl) carbodiimide hydrochloride (EDC) crosslinked immunogens induced lower anti-env IgG titres

To test the difference in responses raised by injection with EDC or non-EDC crosslinked immunogens, responses against all immunogens were compared across groups. There were significantly lower ConM-specific IgG antibodies generated by the EDC-immunogens, (groups B and D) when compared to Group E at 14 days post-second injection (visit 9) (p = 0.001 and p = 0.008, respectively; Table 3). Similarly, a significant difference was observed in the ConS-specific IgG concentrations at this timepoint between groups receiving the EDC-immunogens, Groups B and D compared with Group E (Table 3). After the final injection in Part 1 (Table 1), at 14 days after the third injection (visit 14), significant differences in the ConS-specific antibody titres were maintained between Group B and E (Fig. 2C).

**Table 3:** Summary of Env-specific IgG antibody concentrations post injection.

For IgG responses raised against the stabilised EDC crosslinked immunogens, there were no significant differences observed between any of the groups when comparing the ConM-EDC-specific IgG responses (Fig. 2B; Table 3). However, there were significant differences noted in the IgG responses to ConS-EDC at 14 days after the second injection (visit 9) (Groups A and B v Group E; Table 3).

### Induction of Env-specific memory B cells by ConM and ConS immunogens

**Fig. 3:**
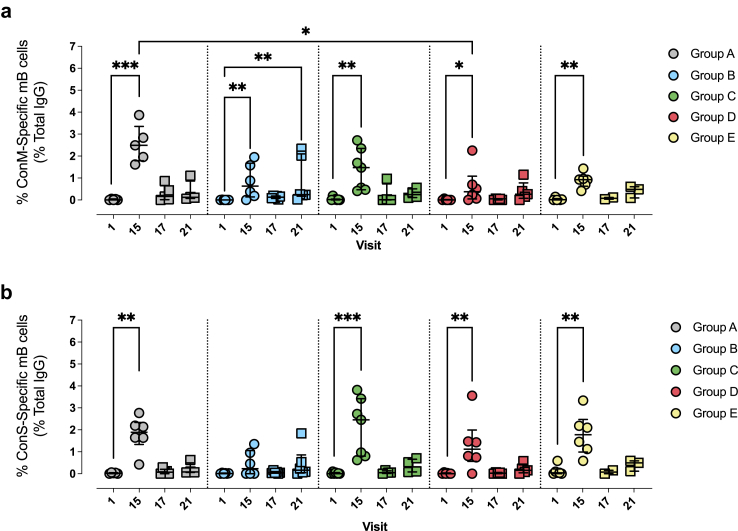
**Memory B cell ELISpot responses to the study-specific Env in participants of the EAVI2020_01 experimental medicine study.** % antigen-specific memory B cells are shown when PBMC from participants of the study were stimulated with either **(a)** ConM or **(b)** ConS. The timepoints assessed were V1 (time of first injection), V15 (28 days post-third IM injection), V17 (time of Mosaic Boost Injection) and V21 (28 days post-Mosaic Boost). In part 1 (circles), Group A (Grey) received three injections with 100 μg ConM, Group B (Light Blue) received three injections with 100 μg ConM-EDC, Group C (Green) received three injections with 100 μg ConS, Group D (Pink) received three injections with 100 μg ConS-EDC and Group E (Yellow) received two injections with 100 μg ConS followed by one injection with 100 μg ConM. All groups were boosted with a cocktail of 50 μg Mosaic 3.1 and 50 μg Mosaic 3.2 (total = 100 μg) during the fourth injection (part 2 (squares)). All injections were adjuvanted with 500 μg MPLA. All data have been background subtracted. Median values with IQR are shown. Kruskal–Wallis Test with Dunn’s multiple correction test was performed to compare the statistical differences between V1 the remaining timepoints within each group, as well as a comparison between groups at V15, V17 and V21. ∗ <0.05; ∗∗<0.01; ∗∗∗ <0.001.

The induction of antigen-specific IgG memory B cells was measured using an ELISpot assay (Fig. 3). A memory B cell response was induced, with cross reactive memory B cells peaking 28 days post 3rd IM injection (visit 15). IgG memory B cells responding to ConM and ConM-EDC were detected at 28 days after the third injection in all groups with median ConM-specific IgG values of 2.50% (Group A), 0.63% (Group B), 1.47% (Group C), 0.38% (Group D) and 0.93% (Group E) and median ConM-EDC-specific IgG values of 0.88% (Group A), 0.55% (Group B), 1.26% (Group C), 0.83% (Group D) and 0.76% (Group E) (Fig. 3A; Supplementary Fig. S1A and Table S3). Similar responses were induced against the ConS and ConS-EDC proteins with median ConS-specific IgG values of 1.90% (Group A), 0.23% (Group B), 2.46% (Group C), 1.12% (Group D) and 1.78% (Group E) and median ConS-EDC-specific IgG values of 1.57% (Group A), 0.54% (Group B), 2.12% (Group C), 2.51% (Group D) and 1.72% (Group E) (Fig. 3B; Supplementary Fig. S1B and Table S3). When comparing across groups at visit 15, only Groups A and D demonstrated a significant difference at this timepoint (p = 0.017), with participants in Group A producing significantly more ConM-specific memory B cells when compared to Group D (Fig. 3A). The antigen-specific memory B cell responses had contracted by the time the mosaic immunogens were given as a fourth injection at visit 17 (part 2), although the interval between injections varied greatly due to schedule interruptions caused by the COVID-19 pandemic (Supplementary Fig. S1). Memory B cell responses against ConM and ConS and ConM-EDC and ConS- EDC were elicited by visit 15, 28 days post the first injection but had waned by visit 17, the time of the Mosaic injection (Fig. 3 and Supplementary Fig. S1). In Group B, there was a significant boost by the Mosaic immunogen against ConM and ConS-EDC (Fig. 3 and Supplementary Fig. S1), and there was a positive trend in the other groups against the other study immunogens (ConM, ConS, ConM-EDC, ConS-EDC, Mos 3.1 and Mos 3.2) after the Mosaic injection (Supplementary Fig. S2 and Table S3).

### Assessment of vaccine induced T cell responses

Vaccine induced T cell responses were assessed by IFN-γ ELISpot (Supplementary Fig. S3). A modest increase in antigen-specific IFN-γ secreting cells 2 weeks post 3rd injection was observed against the consensus peptide pools in Groups C and D, only reaching significance in group D (p = 0.018) (Supplementary Fig. S3A). There was no difference in IFN-γ responses after the mosaic boost (part 2). Furthermore, Intracellular Cytokine Staining used to measure the expression of IFN-γ, TNF-α, IL-2 and CD154 in CD4^+^ and CD8^+^ T cells post stimulation failed to demonstrate any increase in cytokine secreting T cells at any timepoint other than in production of TNF-α in Group B at 2 weeks post-3rd injection (p = 0.032) (Supplementary Figs. S4 and S5). While there was no significant difference over baseline in responses measured by an Activation Induced Marker (AIM) assay, there was a trend for increased CD4^+^ T cell activitation particularly in Group B, at 7 days post the third injection in response to stimulation with both consensus and mosaic peptide pools (Supplementary Fig. S6).

### Neutralising activity of vaccine induced antibodies

**Fig. 4:**
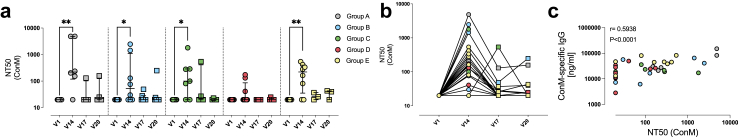
**Virus neutralisation assay responses in serum samples from participants of the EAVI2020_01 experimental medicine study.** Serum samples were tested against **(a)** ConM HIV-1 virus. The timepoints assessed were, Part 1; V1 (time of first injection), V14 (14 days post-third IM injection), Part 2; V17 (time of Mosaic Boost injection) and V20 (14 days post-Mosaic Boost). Serum sample responses shown longitudinally **(b).** These responses were then correlated with ConM-specific IgG serum concentrations **(c)** at V20 (2 weeks post-third injection). In part 1 (circles), Group A (Grey) received three doses of 100 μg ConM, Group B (Light Blue) received three doses of 100 μg ConM-EDC, Group C (Green) received three doses of 100 μg ConS, Group D (Pink) received three doses of 100 μg ConS-EDC and Group E (Yellow) received two doses of 100 μg ConS followed by one dose of 100 μg ConM. All groups were boosted with a cocktail of 50 μg Mosaic 3.1 and 50 μg Mosaic 3.2 during the fourth injection (part 2 (squares)). All injections were adjuvanted with 500 μg MPLA. For **(a)** median values with IQR are shown. Kruskal–Wallis with Dunn’s multiple correction test was performed to compare the statistical differences between V1 the remaining timepoints within each group, as well as a comparison between groups at V14, V17 and V20. ∗ <0.05; ∗∗<0.01. For **(c)**, Spearman’s correlation was performed, and the r and p values are shown within the graphs.

The ability of antibodies raised in participant sera to neutralise the ConS HIV and ConM HIV viruses was measured at four timepoints during the study. The first timepoints were during Part 1 at baseline (visit 1) and two weeks post-third injection of a consensus immunogen (visit 14). The second were at the beginning of Part 2 (visit 17) and at the end of Part 2, two weeks after receiving the final injection of Mos 3.1 + Mos 3.2 (visit 20) (Fig. 4). The sera were tested against ConM and ConS HIV pseudoviruses that were matched to the sequences found in the proteins, as well as a wider panel of global HIV pseudoviruses (Supplementary Table S4), to determine the 50% neutralising titre (NT50) value. Known bNAbs were used as positive controls to demonstrate that ConM and ConS could be neutralised (Supplementary Table S5).

At the end of Part 1, two weeks after receiving the third injection of Env immunogen, a significant increase in the median NT50 was observed against ConM HIV-1 from baseline in Groups A (median NT50 220; 5/6 responders), B (median NT50 53; 5/8 responders), and E (median NT50 222; 6/8 responders), who received ConM SOSIP or EDC ConM SOSIP or ConS (x2) followed by ConM (Fig. 4A) (Group A p = 0.0056; Group B p = 0.0137; Group E p = 0.0031). There was also a significant increase in NT50 in sera from participants in group C (p = 0.0157; median NT50 100; 5/7 responders) who received ConS UFO but not in group D (median NT50 0; 3/8 responders) who received EDC ConS UFO (Fig. 4A).

In those 24 participants who attended for Part 2 of the study, neutralisation activity against ConM and ConS HIV pseudoviruses was not observed at Visit 17, the beginning of Part 2, in most participants except one in Group A, where neutralising function persisted, albeit at a lower level (NT50 130; Fig. 4B). Except for one participant in group B, there was no increase in median NT50 against ConM HIV-1 after the fourth injection with Mos 3.1 and 3.2 in Part 2. Little to no neutralisation activity was observed against ConS HIV-1 in any of the groups (Supplementary Fig. S7A and B). The neutralisation NT50 titres against ConM HIV-1 observed at the end of Part 1 (2 weeks post-third injection) correlated with the IgG concentrations against ConM at the same timepoint (p < 0.0001 and R = 0.5938) (Fig. 4C). No neutralisation was observed in any participant at any timepoint to the wider global panel of HIV-1 pseudoviruses (Supplementary Table S4).

### Electron microscopy-based polyclonal epitope mapping of plasma samples

**Fig. 5:**
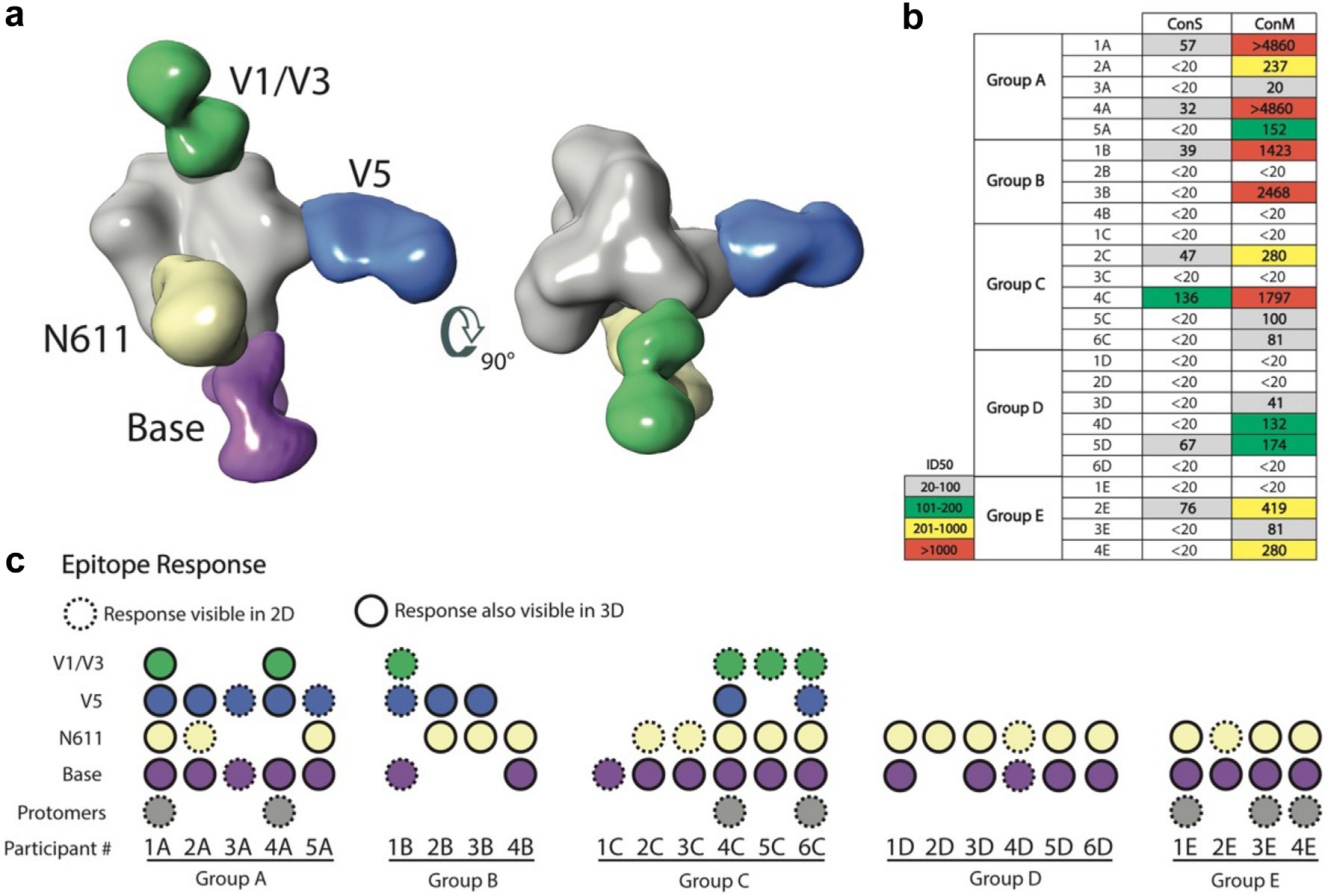
**Electron microscopy-based polyclonal epitope mapping (EMPEM) of plasma samples from participants of the EAVI2020_01 experimental medicine study. (a)** Composite map of HIV-1 trimer with Fabs binding to potential epitopes against V1/V3 (green), V5 (blue), N611 (yellow/beige) and base (purple) regions. **(b)** Summary of ID50 neutralisation results against ConM and ConS HIV-1 trimer. Numbers highlighted in (red) show highest neutralisation response. **(c)** Overview of epitope responses by participants. Dashed circles represent epitopes clearly visible in 2D classes while solid circles represent same epitopes also seen in 3D maps after 3D classification. Some participants had Fab bound to HIV-1 protomers visible in the 2D classes shown in (grey).

Electron Microscopy-based Polyclonal Epitope Mapping (EMPEM) was used to visualise the dominant plasma antibodies and their HIV-1 trimer binding epitopes in plasma samples from the end of Part 1, 2 weeks post third injection (Visit 14). The epitopes mapped included the V1/V3, V5, N611 and base regions of the HIV trimer (Fig. 5A). The strength of binding and the epitopes mapped were then compared to the level of neutralisation observed (Fig. 5B and C). Participants with the most polyclonal antibody response that included the V1/V3 and V5 epitopes tended to have the highest levels of neutralisation (participants 1A, 4A, 1B, 3B and 4C). Conversely, participant samples that predominately mapped to the N611 and base epitopes tended to demonstrate the lowest levels of neutralisation. V1/V3 and V5 epitope antibodies were only visible in groups A, B and C who received ConM SOSIP, EDC ConM SOSIP and ConS UFO, respectively. These results are consistent with preclinical studies showing that the V1V2 domain is involved in the dominant neutralising response induced by ConM SOSIP.

## Discussion

Here we report the results of an experimental medicine study using consensus HIV-1 prefusion-stabilised soluble Env trimers as immunogens to interrogate the human B cell immune response to native-like trimers. The enrolled participants were representative of the local West London, UK, healthy adult population aged 18–55 years and were equally balanced for sex. The pattern of responses was relatively consistent across all assays, with relatively narrow IQR for each group, particularly in the binding antibody responses, that was consistent with the expected variability for induction of vaccine-specific antibody. We believe the findings are generalisable to similar populations.

A limitation of this study was the disruption caused by the COVID-19 pandemic, associated lockdown procedures and COVID-19 research prioritisation. The timing of close-down and re-opening was unrelated to study procedures, and our study population was not in a high-risk category for severe COVID-19, thus the effect on the study is considered to be at random. Despite this disruption, 43/51 participants completed the third (Part 1) and 28/51 participants completed the final injection visit (Part 2). The approximately 50% loss to follow-up rate due to COVID-19 lowered the power of the Part 2 study and limited generalisability of the findings relating to the Mosaic booster immunogens. This may, to some extent, explain the generally non-significant results in Part 2. However, the same trends were detected across the Part 2 participants in all immunological analysis performed, with no outliers, suggesting that the data from the later timepoints are reproducible.

Robust and sustained antigen-binding IgG responses to the different consensus immunogens were observed across all participants and were detectable up to 104 weeks post-third injection. This is encouraging, as vaccine-induced immunity typically wanes quickly over time^44^ and sustained responses can be difficult to induce in the context of HIV-1 Env.^9^ This suggests the likely establishment of bone marrow plasma cells and contrasts with the transient circulation of memory B cells that peaked 4 weeks post third immunisation. The observed durability of antigen-specific binding antibody responses may reflect the activity of the chosen MPLA liposomal adjuvant and dose (500 μg).^45^ While there was limited evidence for the induction of systemic T cell responses following three immunisations in the current study, we have previously reported on the induction of trimer-specific responses in the draining lymph node of vaccinated individuals from this study,^46^ likely critical to driving the observed robust and durable humoral responses. These data obtained here in human participants are consistent with Env-specific B and T cell responses observed in mice administered with the same combination of MPLA liposomes and vaccine antigens.^30^

Modest autologous neutralising antibody responses were detected in participants receiving three injections of ConM trimer. Furthermore, participants that received only ConS UFO trimers also developed neutralising antibody responses against ConM virus demonstrating a degree of cross-reactivity, albeit without neutralising ConS virus. Neutralising antibodies were least frequent in participants that received three injections of the EDC stabilised ConS trimer and appeared highest in the group (E) that received ConS (x2) followed by ConM. The differences between the induction of nAb against ConM and ConS virus is likely due to inherent differences in neutralisation sensitivity between these HIV pseudotype viruses (sequences detailed in Supplementary Table S6). ConS Env is representative of a Tier 1B or Tier 2 virus, whereas ConM is Tier 1A virus.^23^^,^^24^ Neutralisation tier phenotypes correspond to the frequency by which the trimer exists in a closed (tiers 2 and 3), open (tier 1A), or intermediate (tier 1B) conformation. An increasing number of epitopes become exposed as the trimer opens, making the virus more sensitive to neutralisation by certain antibodies^47^ or creating off-target internal epitopes.^48^ Previous studies including our own^42^^,^^43^ have shown that non-native gp120/gp140 vaccines struggle to elicit neutralisation against primary viruses where linear V3 epitopes are occluded. This contrasts to natural infection where autologous neutralisation is often observed within months of primary infection and neutralisation breadth is driven by persistent viremia over a period of years.^4^^,^^5^

EDC cross-linking followed by trimer positive selection using the quaternary epitope-specific bNAb PGT145 as performed in this study, results in well-folded trimers that are ‘locked’ into the ‘closed’ state and retain most bNAb epitopes, particularly in the case of ConS-EDC.^30^ EDC cross-linking prevents trimer opening and would, therefore, be predicted to elicit lower IgG binding titres to Env since reduced internal ‘off-target’ Env epitopes should not be exposed for B cell recognition. This prediction is consistent with the lower Env-specific IgG titres observed in sera from the groups receiving EDC-treated Env (B and D) compared with, for example, group E receiving untreated Env (Fig. 2A, C, D).

EMPEM data indicated a correlation between neutralisation and the induction of antibodies recognising V1V3 and V5 regions of the trimer. Our previous animal studies indicated that neutralising responses to ConM are dominated by specificities targeting the V1V2 region in rabbits^30^ and NHP.^24^^,^^49^^,^^50^ The reduction in serum neutralisation seen with EDC stabilisation most likely reflects masking of V1V2 epitopes by cross-linking within the trimer context, as previously described.^30^ The V1V2 and V5 regions of ConM may present an artificial strain-specific immunodominant epitope, presenting relatively short regions with little conservation across global sequences.^49^ This likely explains why boosting with the two mosaic immunogens that show limited sequence homology to these regions (Supplementary Table S7) failed to maintain autologous ConM neutralisation. Monoclonal antibody isolation will be used to provide further specificity in the mapping of neutralising epitopes from our ongoing phase I trial using the ConM trimer (NCT03961438). Under occupancy of the N-linked glycosylation site, N611, and the non-glycosylated trimer base likely explain the immunodominance of these sites. Immunodominance of the un-glycosylated trimer base was also observed in the recent clinical trial using the prefusion stabilised native like trimer 4571 based on the clade A strain BG505.^1^ However, in this previous study EMPEM did not detect the wider responses to V1V3, V5, and N611 regions observed in the current study. Furthermore, the frequency of autologous neutralising responses against ConM (83%, group A) contrast with the lack of autologous neutralisation seen with trimer 4571 against BG505, although this might reflect in part the neutralisation sensitivity of the parental virus, BG505 being a Tier 2 virus.^1^

As the primary endpoint of this study was to generate neutralising antibodies, it is important to acknowledge that Fc effector function has been shown to contribute to the in vivo antiviral effects of HIV neutralising antibodies.^51^^,^^52^ Furthermore, Fc-mediated effector functions such as antibody-dependent cellular cytotoxicity (ADCC), phagocytosis and complement fixation have been shown to contribute to 25–45% of the total antiviral activity within a non-human primate model.^53^ Future studies should investigate these functions to give a full profile of the antibodies generated by the immunogens used in this study.

The rationale behind the use of consensus immunogens in this study was to minimise strain-specific responses with the aim of improving the chances of eliciting a greater breadth of neutralising antibodies. In this respect, the ConM trimer was chosen based on its ability to bind to the inferred germline precursors of several bNAbs against the trimer apex, albeit with varying affinity.^24^ However, the strain-specific neutralisation and immunodominance of under-glycosylated regions may detract from generating neutralisation breadth. Indeed, the results of this study suggest further design optimisation will be needed to shield the antigenic surfaces promoting strain-specific responses. Although internal non-neutralising epitopes were likely masked by EDC cross-linking, this approach was neither sufficient to block anti-base and anti-N611-associated responses, nor to reveal neutralising responses to alternative conserved group M epitopes. This likely suggests that native trimers alone, even when stabilised with additional chemical cross-links are unlikely to induce neutralising titres of any significant breadth. These observations are in stark contrast to SARS-CoV-2 and respiratory syncytial virus (RSV) where stabilised pre-fusion envelope glycoprotein-based vaccines have resulted in high levels of neutralising antibodies.^54^^,^^55^ This likely reflects the significant challenge posed by the much higher degree of glycan shielding presented by HIV-1 Env compared with these other type 1 fusion proteins. Indeed, while several highly potent HIV-1 bNAbs target the V1V2 region (e.g., PG9, PG16 and PGT145), these are usually dependent on the evolution of long CDRH3 regions required to penetrate the glycan shield surrounding this region. One approach to improve these consensus immunogens would be to lengthen the V1V2 region and increase the glycan density. The immuno-dominance of the non-glycosylated base could also be averted by adding glycosylation or masking through presentation on nanoparticulate structures.^24^

Improved consensus immunogens are still unlikely to induce neutralising antibodies of breadth. Given how bNAbs develop during natural infection in a co-evolutionary process with the virus, where escape viruses trigger renewed rounds of affinity maturation in B cells, eventually leading to bNAbs, it is not surprising that single antigens, even stabilised native-like trimers do not induce bNAbs. It is likely that sequential immunisation starting with a germline-targeting immunogen, followed by “shaping” and “polishing” immunogens, is required for the induction of bNAbs. We propose that the consensus-based native-like trimers that lack isolate specific glycan holes,^53^ may provide suitable immunogens to mature (or “polish”) intermediate responses induced by germline targeting immunogens such as BG505 SOSIP GT1.1^56^^,^^57^ also currently undergoing clinical evaluation (NCT04224701).

## Contributors

R.J.S, K.M.P, H.M.C, R.W.S, G.S, Q.J.S contributed to conception and design. K.M.P was the Chief Investigator. H.M.C coordinated the laboratory data collection and oversight. K.M.P, H.M.C, T.C, M.T, H.L.T, L.R.M, S.D, J.J, K.S, S.Di, I.B, N-M.L, SC, M.Ta and A.B.W contributed to investigation and sample collection. P.M, B.K and D.K provided the immunogens. K.M.P, H.M.C, G.S, R.J.S contributed to data analysis and interpretation. H.M.C, K.M.P and R.J.S wrote the first draft of the paper. All authors critically reviewed and approved the final version of the manuscript. K.M.P, H.M.C, and R.J.S all have assessed and verified the underlying data for this trial.

## Data sharing statement

Raw Immunological data is available in Supplementary material. Additional data may be made available upon requests directed to the corresponding author and after approval of a proposal and with a signed data access agreement. The study protocol and informed consent form are available as Supplementary material.

## Declaration of interests

K.M.P was supported in part by the NIHR Imperial Biomedical Research Centre and by the St Mary’s Development Trust, and has received research funding support from Horizon 2020 and from Trevena Inc, Imperial COVID-19 fund, National Institute for Health Research, and The Sir Joseph Hotung Charitable Settlement outside the submitted work. K.M.P is in receipt of an MRC Clinician Scientist Fellowship award (MR/W024977/1) and a grant from the Chan Zuckerberg Initiative outside the submitted work. K.M.P has received payment or honoraria from CSL Seqirus and Sanofi Pasteur for speaking and as a panellist, and travel support from the Chan Zuckerberg Initiative. K.M.P has participated in data safety monitoring boards for Moderna. K.M.P has a role on the British HIV Association immunisation guidelines writing committee and was on the UK Chief Investigators Group NIHR vaccine research programme.

R.W.S and I.B declare grants or contracts from any entity - EU (EAVI2020; grant number 681137).

B.K. is a co-inventor on a patent covering modified human immunodeficiency virus type 1 (HIV-1) group M consensus envelope glycoproteins (Mosaic) [US-9844589-B2].

A.B.W declares that this work was supported, in whole or in part, by the Bill & Melinda Gates Foundation INV-002916.

All other authors declare no conflict of interests regarding any financial and personal relationships with other people or organisations that could inappropriately influence our work.

## Appendix ASupplementary data

EAVI2020_01 PIS-ICF v7.0

EAVI2020_01_Protocol_v9.0

EAVI2020_Reagents List_SUPPLEMENTAL

EAVI2020_RAW DATA_SUPPLEMENTARY

Supplementary Tables S1-S7

Supplementary Fig. S1 **Figure S1 Memory B Cell ELISpot responses to the study-specific proteins in all participants of the EAVI2020_01 Experimental Medicine Study.** % antigen-specific memory B cells are shown when PBMC from participants of the study were stimulated with either **(a)** ConM-EDC or **(a)** ConS-EDC. The timepoints assessed were V17 (time of Mosaic Boost Injection) and V21 (28 days post-Mosaic Boost). In part 1 (circles), Group A (Grey) received three injections with 100 μg ConM, Group B (Light Blue) received three injections with 100 μg ConM-EDC, Group C (Green) received three injections with 100 μg ConS, Group D (Pink) received three injections with 100 μg ConS-EDC and Group E (Yellow) received two injections with 100 μg ConS followed by one injection with 100 μg ConM. All groups were boosted with a cocktail of 50 μg Mosaic 3.1 and 50 μg Mosaic 3.2 during the fourth injection (part 2 (squares)). All injections were adjuvanted with 500 μg MPLA. All data have been background subtracted. Median values with IQR are shown. Mann-Whitney Test was performed to compare the statistical differences between V17 and V21 within each group, as well as a Kruskal-Wallis Test with Dunn’s multiple comparisons between groups at V17 and V21.

Supplementary Fig. S2 **Figure S2 Memory B Cell ELISpot responses to the study-specific proteins in all participants of the EAVI2020_01 Experimental Medicine Study.** % antigen-specific memory B cells are shown when PBMC from participants of the study were stimulated with either **(a)** Mos 3.1 or **(b)** Mos 3.2. The timepoints assessed were V17 (time of Mosaic Boost Injection) and V21 (28 days post-Mosaic Boost). In part 1, Group A (Grey) received three injections with 100 μg ConM, Group B (Light Blue) received three injections with 100 μg ConM-EDC, Group C (Green) received three injections with 100 μg ConS, Group D (Pink) received three injections with 100 μg ConS-EDC and Group E (Yellow) received two injections with 100 μg ConS followed by one injection with 100 μg ConM. All groups were boosted with a cocktail of 50 μg Mosaic 3.1 and 50 μg Mosaic 3.2 during the fourth injection (part 2 (squares)). All injections were adjuvanted with 500 μg MPLA. All data have been background subtracted. Median values with IQR are shown. Mann-Whitney Test was performed to compare the statistical differences between V17 and V21 within each group, as well as a Kruskal-Wallis Test with Dunn’s multiple comparisons between groups at V17 and V21.

Supplementary Fig. S3 **Figure S3 IFN-g ELISpot responses to the study-specific peptide pools in all participants of the EAVI2020_01 experimental medicine study.** Spot Forming Units (SFU) per million cells are shown when PBMC participants of the study were stimulated with either **(a)** Consensus Peptide Pools, or **(b)** Mosaic Peptide Pools. The timepoints assessed were V1 (time of first injection), V14 (14 days post-third IM injection), V17 (time of Mosaic Boost Injection) and V20 (14 days post-Mosaic Boost). In part 1 (circles), Group A (Grey) received three injections with 100 μg ConM, Group B (Light Blue) received three injections with 100 μg ConM-EDC, Group C (Green) received three injections with 100 μg ConS, Group D (Pink) received three injections with 100 μg ConS-EDC and Group E (Yellow) received two injections with 100 μg ConS followed by one injection with 100 μg ConM. All groups were boosted with a cocktail of 50 μg Mosaic 3.1 and 50 μg Mosaic 3.2 during the fourth injection (part 2 (squares)). All injections were adjuvanted with 500 μg MPLA. All data have been background subtracted and the line at 55 SFU/million cells represents the cutoff for positivity. Median values with IQR are shown. The Kruskal-Wallis with Dunn’s multiple correction test was performed to compare the statistical differences between V1 the remaining timepoints within each group, as well as a comparison between groups at V14, V17 and V20. ∗ <0.05.

Supplementary Fig. S4 **Figure S4 Intracellular Cytokine Staining (ICS) responses to the study-specific Consensus peptide pools in PBMC from participants of the EAVI2020_01 experimental medicine study.** CD4^+^ IFN- γ **(a)**, TNF-α **(b)**, IL-2 **(c)**, CD154 **(d)** and CD8^+^ IFN-γ **(e)**, TNF-α **(f)**, IL-2 **(g)**, CD154 **(h)** responses are shown as % of live parent. The timepoints assessed were V1 (time of first injection), V14 (14 days post-third IM injection), V17 (time of Mosaic Boost Injection) and V20 (14 days post-Mosaic Boost). In part 1 (circles), Group A (Grey) received three injections with 100 μg ConM, Group B (Light Blue) received three injections with 100 μg ConM-EDC, Group C (Green) received three injections with 100 μg ConS, Group D (Pink) received three injections with 100 μg ConS-EDC and Group E (Yellow) received two injections with 100 μg ConS followed by one injection with 100 μg ConM. All groups were boosted with a cocktail of 50 μg Mosaic 3.1 and 50 μg Mosaic 3.2 during the fourth injection (part 2 (squares)). All injections were adjuvanted with 500 μg MPLA. All data have been background subtracted. Median values with IQR are shown. The Kruskal-Wallis with Dunn’s multiple correction test was performed to compare the statistical differences between V1 the remaining timepoints within each group, as well as a comparison between groups at V14, V17 and V20. ∗ <0.05.

Supplementary Fig. S5 **Figure S5 Intracellular Cytokine Staining (ICS) responses to the study-specific Mosaic peptide pools in PBMC from participants of the EAVI2020_01 experimental medicine study.** CD4^+^ IFN- γ **(a)**, TNF-α **(b)**, IL-2 **(c)**, CD154 **(d)** and CD8^+^ IFN-γ **(e)**, TNF-α **(f)**, IL-2 **(g)**, CD154 **(h)** responses are shown as % of live parent. The timepoints assessed were V1 (time of first injection), V14 (14 days post-third IM injection), V17 (time of Mosaic Boost Injection) and V20 (14 days post-Mosaic Boost). In part 1 (circles), Group A (Grey) received three injections with 100 μg ConM, Group B (Light Blue) received three injections with 100 μg ConM-EDC, Group C (Green) received three injections with 100 μg ConS, Group D (Pink) received three injections with 100 μg ConS-EDC and Group E (Yellow) received two injections with 100 μg ConS followed by one injection with 100 μg ConM. All groups were boosted with a cocktail of 50 μg Mosaic 3.1 and 50 μg Mosaic 3.2 during the fourth injection (part 2 (squares)). All injections were adjuvanted with 500 μg MPLA. All data have been background subtracted. Median values with IQR are shown. The Kruskal-Wallis with Dunn’s multiple correction test was performed to compare the statistical differences between V1 the remaining timepoints within each group, as well as a comparison between groups at V14, V17 and V20.

Supplementary Fig. S6 **Figure S6 Activation Induced Assay (AIM) responses to the study-specific peptide pools in PBMC from participants of the EAVI2020_01 experimental medicine study.** CD4^+^ CD69+CD40L+ are shown in response to **(a)** Consensus Peptide Pools 1 and 2 or **(b)** Mosaic Peptide Pools 1 and 2. The timepoints assessed were V1 (time of first injection), V13 (7 days post-third IM injection), V17 (time of Mosaic Boost Injection) and V20 (14 days post-Mosaic Boost). In part 1 (circles), Group A (Grey) received three injections with 100 μg ConM, Group B (Light Blue) received three injections with 100 μg ConM-EDC, Group C (Green) received three injections with 100 μg ConS, Group D (Pink) received three injections with 100 μg ConS-EDC and Group E (Yellow) received two injections with 100 μg ConS followed by one injection with 100 μg ConM. All groups were boosted with a cocktail of 50 μg Mosaic 3.1 and 50 μg Mosaic 3.2 during the fourth injection (part 2 (squares)). All injections were adjuvanted with 500 μg MPLA. Median values with IQR are shown. The Kruskal-Wallis with Dunn’s multiple correction test was performed to compare the statistical differences between V1 the remaining timepoints within each group, as well as a comparison between groups at V14, V17 and V20.

Supplementary Fig. S7 **Figure S7 virus neutralisation assay responses in serum samples from participants of the eavi2020_01 experimental medicine study.** Serum samples were tested against **(a)** ConS HIV-1 virus. The timepoints assessed were V1 (time of first infection), V14 (14 days post-third IM injection), V17 (time of Mosaic Boost injection) and V20 (14 days post-Mosaic Boost). These responses were then compared to ConS-specific IgG serum concentrations **(b)** at V20 (2 weeks post-third injection). In part 1 (circles), Group A (Grey) received three doses of 100 mg ConM, Group B (Light Blue) received three doses of 100 μg ConM-EDC, Group C (Green) received three doses of 100 μg ConS, Group D (Pink) received three doses of 100 μg ConS-EDC and Group E (Yellow) received two doses of 100 μg ConS followed by one doses of 100 μg ConM. All groups were boosted with a cocktail of 50 μg Mosaic 3.1 and 50 μg Mosaic 3.2 during the fourth injection (part 2 (squares)). All injections were adjuvanted with 500 μg MPLA. For **(a)** median values with IQR are shown. The Kruskal-Wallis with Dunn’s multiple correction test was performed to compare the statistical differences between V1 the remaining timepoints within each group, as well as a comparison between groups at V14, V17 and V20. ∗ <0.05; ∗∗<0.01. For **(b)**, Spearman’s correlation was performed, and the r and p values are shown within the graphs.
