# Supplementary material for: Experimental medicine study with stabilised native-like HIV-1 Env immunogens drives long-term antibody responses, but lacks neutralising breadth
Source: eBioMedicine. 2025 Jan 2;112:105544. doi: 10.1016/j.ebiom.2024.105544 (PMC11753977; doi:10.1016/j.ebiom.2024.105544)
Supplement: EAVI2020_01 PIS-ICF v7.0 [file mmc1.pdf]

Study Title: An experimental medicine study modelling the interaction between rationally-designed synthetic model viral protein immunogens and the breadth of the induced B and T cell repertoires.

Study ID: EAVI2020_01 **IRAS ID: 251930** **Participant Information Sheet**

Invitation:

- We invite you to participate in an experimental medicine study
- Before you decide to take part, it is important for you to understand why the study is being done and what it involves
- Please take time to read the following information carefully
- You are free to decide if you want to take part. Just ask us if there is anything that is not clear or if you would like more information

Contents:

1: What is the purpose of the study?

2: Who can take part?

3: What would taking part involve?

4: What are the possible benefits?

5: What are the possible disadvantages and risks?

6: Supporting information

7: Consent form

Brief Summary:

- We want to investigate how administering different combinations of specially designed proteins influences our body's immune response to those proteins
- You will be in the study for 7–12 months, making up to 21 scheduled visits to the Imperial Clinical Research Facility, Hammersmith Hospital in London
- There will be 3 or 4 visits when you will be injected with proteins into the muscle of your arm
- You will be paid for your time, travel and inconvenience: £100 per completed visit

Contact:

If you have any questions about this study, please talk to the lead researcher:

Dr Katrina Pollock
Tel 020 3313 8070

1. What is the Purpose of the Study?

The immune system can produce antibodies and white blood cells as a protective response against foreign proteins. This is how vaccines work to protect against infection. We have designed some "model" proteins, based on natural proteins, to investigate how the body responds to specific protein shapes and combinations. We will assign you to one of the study groups to receive a different combination of the model proteins. By doing this we hope to better understand

how the body's defence mechanisms against infection work.

The proteins being used in this study are known as: ConM SOSIP, EDC ConM SOSIP, ConS UFO, EDC ConS UFO, Mosaic SOSIPs Mos3.1, Mos3.2 and Mos3.3, 763 SOSIP, and AMC011. They are all synthetic (manufactured in a factory) and pose no risk of infection. They are very subtle variations of naturally occurring proteins produced by viruses, in this case HIV virus. We selected these because similar proteins have been given to many people without safety concerns, and we can measure the body's responses to them with great precision. **ConM SOSIP and ConS UFO** are two different versions of the protein which forms a shell around the HIV virus. **EDC ConM SOSIP and EDC ConS UFO** are versions of those proteins that have been chemically treated to make them more rigid in shape. **Mosaic SOSIPs Mos3.1, Mos3.2 and Mos3.3** are three HIV shell proteins that have been designed using computers. **AMC011 and 763 SOSIP** are HIV shell proteins which we hope will result in antibodies which can neutralise many strains of HIV. We predict that using combinations of these immunogens will give a better immune response than using just one.

In addition we will add an oil and water mixture ("MPLA") that has been widely used to boost immune responses. We have tested our ConM and ConS proteins, in combination with MPLA, in an animal model and observed a complete lack of toxic effects.

2. Who Can Take Part?

You can take part only if you give your consent after being fully informed about what participation involves. A study doctor/nurse will discuss this written information with you.

There are strict eligibility criteria for participation:

- You must be healthy and aged 18–55 years
- You must be available during a 7- to 12-month period to attend up to 21 scheduled visits, 3 or 4 of which will involve you receiving an injection in your arm with a model protein
- You must be able to read and speak English well enough to understand the procedures required and risks and benefits
- If you are a female of childbearing potential you must test negative for pregnancy and, if heterosexually active, must agree to use hormonal contraception, or an IUD (intrauterine device), until 12 weeks after your final injection

You will not be able to take part if you:

- Are pregnant or breast feeding

- Have a significant history of allergy
- Are immunocompromised (your body's immune system is not working fully) or HIV infected at the point of screening. We will also exclude people who may be at high risk of catching HIV.

There may be other reasons why you cannot take part – if so the study doctor/nurse will discuss these with you.

At the end of the consenting process, which usually takes about 1 hour, the study doctor/nurse will ask you to sign a consent form.

3. What would taking Part Involve?

All visits will take place at the NIHR Imperial Clinical Research Facility (Imperial CRF) at Hammersmith Hospital, Du Cane Road, London W12 0HS. We will register you as a patient of Imperial College Healthcare NHS Trust before being consented to participate in the study.

Every visit is very important to the study, so please agree to take part only if you genuinely expect to be able to attend them all.

Participants will be allocated by a random process to one of the study groups. The staff caring for you will know which group you are in, but you will not. Each group will receive a different combination of model proteins, although the total amount per injection will be the same for each group/participant.

Screening visit

After you have given your consent, we will screen you to ensure that you satisfy all of the study's eligibility criteria. Screening will take about two hours, and includes:

- A discussion about medical history, including medication
- A symptom-directed physical examination
- Height, weight, blood pressure, heart rate and oral temperature measurements
- Blood samples to check your health, HIV antibody status and urine samples for pregnancy (women only)

Injection visits

Injection visits will last about 3 hours. Before you are injected, we will do a basic health check (symptoms, medication, symptom-directed physical exam [if required], blood pressure, heart rate and oral temperature), do a urine pregnancy test (women only) and collect blood samples. Some of these blood samples are relevant for participants randomized to one of the study groups only. After the

injection you will be observed for one hour for any immediate reaction to the protein.

For some participants (depending on which group you are assigned to), the second, third and fourth injection visits will be 84, 168 and 336 days after the first injection. For participants in the other groups they will be 56 and 112 (and 168 if applicable) days after the first injection. They will be the same as the first injection visit.

Follow-up visits, blood samples and lymph node sampling

The pattern of follow-up visits after injection depends on the study group to which you are assigned. All participants will attend the hospital for blood tests **at 7, 14, and 28 days** after each injection, and some will also attend **1 and 84 days** after some injections. These visits will last about 1h. Visits must take place on the indicated days.

The participants in some groups also have the option to donate a lymph node sample on 3 occasions. This may involve a visit to a different hospital in London, and you will receive an additional £100 for each donation. The procedure is described in a separate information sheet, with a separate consent form. There are some additional eligibility criteria for this procedure. This procedure is optional and deciding not to take part doesn't affect your participation in the main study.

The visit schedules are summarised in the tables below.

COVID-19

Restarting participation following a pause due to COVID-19

If we had to pause your participation due to the COVID-19 pandemic, we will restart you at your next scheduled immunogen challenge visit. However, in order to check that your health status hasn't changed since we last saw you, you will attend the Imperial CRF for a blood sample a few days before the challenge visit. You will be paid for the extra visit, as described in Section 4 below.

Receipt of vaccines outside the study schedule including authorised COVID-19 vaccines

If you are offered an authorised COVID-19 vaccine, we encourage you to have it. We request that you have it at least 14 days before or after immunogen challenge in the study, and at least 28 days if possible. For seasonal influenza vaccine we request at least 7 days before or after immunogen challenge in the study, and at least 28 days if possible. We request that you have these vaccines

in the opposite arm from the study injection.

Your safety during COVID-19

During your study visits you will be asked to adhere to public health guidelines against COVID-19. These may include maintaining social distancing, wearing face masks, and hand hygiene. If you become unwell and suspect that you may have COVID-19, please inform the study team before attending any visit.

Schedule for Groups A–E

Visit	Screen	1	2	3	4	5	6	7	8	9	10	11	12	13	14	15	16	17	18	19	20	21
Day		0	1	7	14	28	84	85	91	98	112	168	169	175	182	196	252	336	337	343	350	364
Vist Window (Days)		0	0	−1 to +1	−2 to +2	−2 to +2	−7 to +14	0	−1 to +1	−2 to +2	−2 to +2	−7 to +14	0	−1 to +1	−1 to +1	−2 to +2	−2 to +2	−7 to +14	0	−1 to +1	−2 to +2	−2 to +2
Immunogen Challenge		✓					✓					✓						✓				
Vital Signs	✓	✓					✓					✓						✓				
Blood sample (all groups)	✓	✓		✓	✓	✓	✓		✓	✓	✓	✓		✓	✓	✓	✓	✓	✓	✓	✓	✓
Extra blood sample Group A only		✓	✓	✓			✓	✓	✓			✓	✓	✓								
Optional Lymph node sample (Groups B-E only)		✓			✓					✓												
Urine pregnancy	✓	✓					✓					✓						✓				

Schedule for Groups F–I

Visit	Screen	1	2	3	4	5	6	7	8	9	10	11	12	13	14	15	16	17
Day		0	7	14	28	56	63	70	84	112	119	126	140	168	175	182	196	252
Visit Window (Days)		0	−1 to +1	−2 to +2	−2 to +2	−7 to +14	−1 to +1	−2 to +2	−2 to +2	−7 to +14-	−1 to +1	−2 to +2	−2 to +2	−7 to +14-	−1 to +1	−2 to +2	−2 to +2	−7 to +14
Immunogen Challenge		✓				✓				✓				✓				
Vital Signs	✓	✓				✓				✓				✓				
Blood sample	✓	✓	✓	✓	✓	✓	✓	✓	✓	✓	✓	✓	✓	✓	✓	✓	✓	✓
Optional lymph node sample		✓		✓				✓										
Urine pregnancy	✓	✓				✓				✓				✓				

Schedule for Groups J–M

Visit	Screen	1	2	3	4	5	6	7	8	9	10	11	12	13
Day		0	7	14	28	56	63	70	84	112	119	126	140	196
Visit Window (Days)		0	−1 to +1	−2 to +2	−2 to +2	−7 to +14	−1 to +1	−2 to +2	−2 to +2	−7 to +14	−1 to +1	−2 to +2	−2 to +2	−7 to +14
Immunogen Challenge		✓				✓				✓				
Vital Signs	✓	✓				✓				✓				
Blood sample	✓	✓	✓	✓	✓	✓	✓	✓	✓	✓	✓	✓	✓	✓
Optional lymph node sample		✓		✓				✓						
Urine pregnancy	✓	✓				✓				✓				

Total blood draw

Over the course of the whole study we expect to take a total of approximately 530–980 millilitres (about 1–1¾ pints) of blood, depending on which group you are assigned to. For participants who restart their participation following a pause due to COVID-19, up to 50 millilitres more blood may be taken.

4. What are the Possible Benefits?

There will be no known health benefit from participating in this study. We will pay you for your time, inconvenience and travel expenses; £100 per completed visit, £100 per lymph node sample. You will receive £100 for any extra visit required by the study team. You will receive a lump sum of at the end of your participation for completed visits. As a bonus, you will receive an additional £100 if you attend all of your study visits on time and comply with all study requirements. If you are withdrawn from the study by the Principal Investigator for medical reasons you will receive full reimbursement.

Participants who attend screening but who are not enrolled will not receive payment.

5. What are the Possible Disadvantages and Risks?

No serious common reactions are anticipated from the model proteins or MPLA. Various (non-serious) reactions associated with injection of similar proteins and with MPLA may include short-lived: pain, tenderness, warmth, redness, itching and swelling at the injection site, flu-like symptoms, general muscle aches, tiredness, headache and nasal congestion.

Rarely, a serious allergic reaction may occur.

Fainting may also occur around the time of injection or blood sampling, particularly if you strongly dislike needles. We'll minimise the risk by asking you to recline or lie down during those procedures.

Blood draw can sometimes cause pain, and bruising and soreness of the arms, and can very rarely result in blockage of a vein or a small nerve injury which can cause numbness. Normally these problems disappear with time.

The model proteins have not been tested for safety in pregnancy. Therefore you must not become pregnant during the study period up to 12 weeks after your final injection. If you find out that you have become pregnant during the study, you must tell the study team immediately.

Because the model proteins are based on naturally occurring HIV proteins you may develop antibodies that react in blood tests for HIV infection. This means that you may test positive for HIV infection even if you don't have it (a false

positive). However, there are tests available which can demonstrate your true HIV status. We will also provide you with a letter explaining your participation in this study and we can advise the laboratories on how to proceed in this situation. However, this may prevent you from donating blood or blood products until these antibodies disappear naturally which may take some years. Insurance companies only require applicants to declare actual HIV infection, and so having antibodies against HIV should not affect insurance.

Administration of these model proteins will not provide protection against infection with HIV. We do not know what effect any antibodies you may develop against the model proteins could have on the course of infection if you did get infected with HIV in the future. Participants should therefore practice normal "safe sex" such as using condoms to prevent sexually transmitted infections. We will also exclude persons who are at high risk of being infected with HIV.

We will provide support for you in the case that any of the screening tests we do may detect anything that requires further medical intervention. We can, with your permission, contact your GP or other specialised services to follow-up any abnormal findings such as a positive HIV or hepatitis test.

6. Supporting Information

What will happen if I don't want to carry on with the study?

Participation is voluntary and you are free to withdraw at any time without giving any reason (although we will usually ask you why), without your medical care or legal rights being affected.

What will happen to the results of this study?

We hope to publish the results in open-access peer-reviewed medical journals, and present them to the wider scientific community at international conferences. You will not be named in any of these or identified in any other way. The results and description of the study will also be available on the public website www.clinicaltrials.gov.

What happens if the study stops early?

If the study is stopped early you will be paid for the number of scheduled visits you've completed, and the reasons for stopping the study will be fully explained to you.

What if something goes wrong?

The Imperial Clinical Research Facility is equipped and staffed to manage very high risk clinical trials, and is part of the Hammersmith Hospital. We can therefore

respond to and manage any emergencies that may unexpectedly arise during our studies and trials.

If you feel you have been harmed by taking part in this study you should discuss this with the team looking after you. They will advise you on the likelihood of the study itself causing the problem.

Imperial College London holds insurance policies which apply to this study. If you experience harm or injury as a result of taking part in this study, you will be eligible to claim compensation without having to prove that Imperial College is at fault. This does not affect your legal rights to seek compensation.

If you are harmed due to someone's negligence, then you may have grounds for a legal action. If you are still not satisfied with the response, you may contact the Imperial College, Joint Research Compliance Office.

What if I have a complaint?

If you wish to complain, or have any concerns about any aspect of the way you have been treated during the course of this study, please inform the lead researcher:

- **Dr Katrina Pollock: Tel 0203 313 8070**

If you'd prefer to speak to someone independent of the study, the normal NHS complaint mechanisms are also available to you:

- Patient Advice and Liaison Service (PALS): Tel 020 3313 0088 (Mon–Fri 9am–5pm), or email pals@imperial.nhs.uk

Will my taking part in this study be kept confidential?

Yes. Your data and samples will be anonymised before being sent for analysis to our internal laboratories. Your name will not be disclosed outside the host NHS site, and only the delegated study team will have your full details. Your GP will be notified if you participate in the experimental study. To ensure the study is properly conducted your data may be looked at by persons authorised by the study sponsor to carry out study monitoring or auditing. All anonymised data generated from the results of this study will be stored on Imperial College London servers for 10 years.

If it arises that a participant who has given informed consent, loses capacity to consent during the study, the participant and all identifiable data or tissue collected will be withdrawn from the study. Data or tissue which is not identifiable to the research team may be retained.

A description of the study will also be available on the public website

clinicaltrials.gov. This website will not include information that will identify you. You will be able to access a summary of the results via the website when the study has been completed. You can ask a member of staff for more details about which website the information is available on.

Data will be analysed at a number of sites both within and outside the UK and also within the EAVI2020 consortium. Data generated from the study will be used to formulate a research paper of the results. This paper will document our findings and discussions based on the results generated from the study.

Your details will be entered on The Over-volunteering Prevention System (TOPS), which aims to prevent participants from taking part too frequently in clinical research studies. This is not a public database, and can only be accessed by various registered clinical sites.

General Data Protection Regulation (GDPR)

Imperial College London is the sponsor for this study based in the United Kingdom. We will be using information from you in order to undertake this study and will act as the data controller for this study. This means that we are responsible for looking after your information and using it properly. Imperial College London will keep identifiable information about you for 10 years after the study has finished in relation to data subject consent forms.

Further information on Imperial College London's retention periods may be found at https://www.imperial.ac.uk/media/imperial-college/administration-and-support-services/records-and-archives/public/RetentionSchedule.pdf.

Your rights to access, change or move your information are limited, as we need to manage your information in specific ways in order for the research to be reliable and accurate. If you withdraw from the study, we will keep the information about you that we have already obtained. To safeguard your rights, we will use the minimum personally-identifiable information possible. You can find out more about how we use your information from the study PI, Dr Katrina Pollock.

Legal Basis

As a university we use personally-identifiable information to conduct research to improve health, care and services. As a publicly-funded organisation, we have to ensure that it is in the public interest when we use personally-identifiable information from people who have agreed to take part in research. This means that when you agree to take part in a research study, we will use your data in the ways needed to conduct and analyse the research study.

Health and care research should serve the public interest, which means that we have to demonstrate that our research serves the interests of society as a whole. We do this by following the UK Policy Framework for Health and Social Care Research.

International Transfers

There may be a requirement to transfer information to countries outside the European Economic Area (for example, to a research partner). Where this information contains your personal data, Imperial College London will ensure that it is transferred in accordance with data protection legislation. If the data is transferred to a country which is not subject to a European Commission (EC) adequacy decision in respect of its data protection standards, Imperial College London will enter into a data sharing agreement with the recipient organisation that incorporates EC approved standard contractual clauses that safeguard how your personal data is processed.

Contact Us

If you wish to raise a complaint on how we have handled your personal data or if you want to find out more about how we use your information, please contact Imperial College London's Data Protection Officer via email at dpo@imperial.ac.uk, via telephone on 020 7594 3502 and via post at Imperial College London, Data Protection Officer, Faculty Building Level 4, London SW7 2AZ.

If you are not satisfied with our response or believe we are processing your personal data in a way that is not lawful you can complain to the Information Commissioner's Office (ICO). The ICO does recommend that you seek to resolve matters with the data controller (us) first before involving the regulator.

Imperial Clinical Research Facility (ICRF) will collect information from you for this research study in accordance with our instructions. The ICRF will use your name, and contact details to contact you about the research study, and make sure that relevant information about the study is recorded for your care, and to oversee the quality of the study. Individuals from Imperial College London and regulatory organisations may look at your research records to check the accuracy of the research study. The ICRF will pass these details to Imperial College London along with the information collected from you. The only people in Imperial College London who will have access to information that identifies you will be people who need to contact you to discuss your results or the study, or audit the data collection process. The people who analyse the information will not be able to identify you and will not be able to find out your name, or contact details. The ICRF will keep identifiable information about you from this study for 10 years after the study has finished.

What will happen to any samples I give?

As part of the study, some samples will be tested at the host NHS site, and some at Imperial College London. Other samples might be sent from Imperial College London to other labs both inside and outside of the UK. We will only be sending samples to our consortium collaborators (EAVI2020). These labs won't have access to your personal identifiable information, and will also be blinded to your treatment group and regime. Samples leftover at the end of the study will be transferred to a tissue bank and kept for up to 10 years, for use in future research.

Who is organising and funding the study?

The study is organised by Imperial College London, UK. The study is funded by the European Union; Horizon 2020. The host NHS site is Imperial College Healthcare NHS Trust, of which Hammersmith Hospital is a part.

Who has reviewed the study?

This study has been reviewed and approved by the London - Fulham Research Ethics Committee, the Health Research Authority, and by the Research & Development office of the host NHS site.

Contacts during the study, including emergencies

You can contact the study nurses and doctors on Tel 020 3313 1703 (Mon–Fri 9am–5pm).

For **emergencies** outside those hours, please call 07826 903 646 and tell the person who answers that you are a participant in the EAVI2020 study at the Imperial CRF, and the nature of the emergency; a call-back will be arranged. Alternatively, please dial 111, visit your GP or the nearest NHS Drop-In or Accident and Emergency Department if you have any medical concerns.

Thank you for your interest in taking part in this study. If you choose to proceed to the screening assessments, you will be given a copy of this information sheet and your signed consent form to keep.

7. Consent Form

Subject ID: ____________

Study Title: An experimental medicine study modelling the interaction between rationally-designed synthetic model viral protein immunogens and the breadth of the induced B and T cell repertoires.

Study ID: EAVI2020_01 **IRAS ID: 251930** **PI: Dr Katrina Pollock**

	**Write initials in box**
I confirm that I have read the information sheet for the above study. I have had the opportunity to consider the information, ask questions and have had these answered satisfactorily.	☐
I understand that my participation is voluntary and that I am free to withdraw at any time without giving any reason, without my medical care or legal rights being affected.	☐
I understand that my relevant medical history, and data collected during the study, may be looked at by individuals from Imperial College London or from the host NHS Trust, where it is relevant to my taking part in this study. I give permission for these individuals to have access to my records.	☐
I give permission for my samples and data to be analysed, at various sites within and outside the UK, and the results published in a way that doesn't identify me.	☐
I agree to my General Practitioner being informed of my participation in the study, and to being entered onto The Over-Volunteering Prevention System (TOPS).	☐
I give permission for my samples to be stored for ethically approved studies after the end of this study.	☐
I agree to take part in the study.	☐

____________	____________	____________
Name of participant	Date	Signature
Name of person taking consent	Date	Signature
