# Supplementary material for: Experimental medicine study with stabilised native-like HIV-1 Env immunogens drives long-term antibody responses, but lacks neutralising breadth
Source: eBioMedicine. 2025 Jan 2;112:105544. doi: 10.1016/j.ebiom.2024.105544 (PMC11753977; doi:10.1016/j.ebiom.2024.105544)
Supplement: EAVI2020_01_Protocol_v9.0 [file mmc2.pdf]

Study Protocol Title

An experimental medicine study modelling the interaction between rationally-designed synthetic model viral protein immunogens and the breadth of the induced B and T cell repertoires.

Investigators

Dr Katrina Pollock – Chief Investigator: Clinical activities will be carried out at the NIHR Imperial CRF, Hammersmith Hospital, Imperial College Healthcare NHS Trust.

Professor Robin Shattock – Scientific Co-Investigator: Immunogenicity laboratory investigations will be performed in the Department of Infection and Immunity at Imperial College London.

Sponsor

Imperial College London will act as the main Sponsor for this study. Delegated responsibilities will be assigned to Imperial College Healthcare NHS Trust as necessary.

Manufacturer of Immunogens

Polymun Scientific
Immunbiologische Forschung GmbH
Donaustr
99, 3400 Klosterneuburg
Austria
www.polymun.com

Protocol Version: 9.0

Protocol Date: 25 Jan 2022

THE CONFIDENTIAL INFORMATION IN THIS DOCUMENT IS PROVIDED TO YOU AS AN INVESTIGATOR, POTENTIAL INVESTIGATOR, OR CONSULTANT, FOR REVIEW BY YOU, YOUR STAFF, AND APPLICABLE INSTITUTIONAL REVIEW BOARDS (IRBs) AND/OR INDEPENDENT ETHICS COMMITTEES (IECs). IT IS UNDERSTOOD THAT THE INFORMATION WILL NOT BE DISCLOSED TO OTHERS, EXCEPT TO THE EXTENT NECESSARY TO OBTAIN ETHICAL AND REGULATORY APPROVAL FROM THE RESPECTIVE COMMITTEE'S AGENCIES AND INFORMED CONSENT FROM THOSE PERSONS TO WHOM THE IMMUNOGENS MAY BE ADMINISTERED.

Background

One of the most effective arms of the human immune system is the ability of very low concentrations of antibody proteins to bind to viruses, bacteria and toxins and "neutralise" their activity or ability to infect. In contrast to cellular immunity, which may cause tissue destruction and pathology, antibody-mediated immunity can be very passive, while completely preventing infection. How antibodies bind their targets varies enormously, ranging from unhelpful "blocking" antibodies or narrowly focussed neutralising antibodies, to highly protective "broadly neutralising" antibodies (bNAbs) that can neutralise a wide range of strains of the same pathogen. Such bNAbs are especially sought after in virus infections such as HIV, influenza and others where the virus mutates to evade immune responses that are too narrow or focussed. Antibodies arise when an "immunogen" (an immunogen is anything that induces an immune response, typically a foreign protein) is taken up by the immune system and shown to white blood cells – T and B cells - by specialised immune cells. In some cases the T and B cells bind the immunogens to receptors on their surface, triggering an immune response in which T cells "help" B cells to manufacture specific antibodies. The events around how the protein is processed into manageable pieces, shown to the T and B cells, and the pattern of chemical signals produced by the immune cells is highly complex, but eventually determines how broad the antibody response will be (its breadth). For infections like HIV and influenza, decades of research and clinical vaccine trials have had limited or no success. To take HIV as an example, we have an almost complete lack of understanding of how immunogens interact with the naive human B cell receptor (BCR) repertoire and the pathways required to induce bNAbs during an infection or after an immunisation. Animal models have failed as the naïve, germline encoded, B cell antibody receptor repertoires of non-human species are sufficiently different from those of humans to render design and selection of vaccines based on non-human species problematic. Additionally, bNAbs isolated from HIV-1-infected individuals have structural features that occur rarely or not at all in other mammals, such as unusually long loop-binding regions (CDRH3 loops) required to penetrate past glycans on the surface of the envelope spike that shield key neutralising epitopes (Mascola JR 2013). There is therefore a critical need to better understand, in human experimental medicine models of immune challenge, how immunogens and B/T cells interact in the development of protective bNAb anti-viral responses.

Our approach to resolving this impasse is to challenge the human immune system with rationally-designed model immunogens to determine the structural and other characteristics required to drive human B cell antibody responses towards neutralisation breadth. We have selected HIV as an experimental model as there is a reasonable understanding about the specificity and function of anti-HIV bNAbs, as well as an urgent need to identify novel immunisation approaches following decades of failed or poorly successful trials. There is also a huge database of safety using HIV proteins as immunogens, and the technological expertise to design and manufacture HIV viral proteins. Assays for HIV neutralising activity are also well established in our laboratories. Although focussed on HIV, our findings will be applicable to other viral infections.

The model immunogens we propose to use in these experimental medicine studies are unlikely to be suitable as vaccines, and any clinical development would require iterative cycles of design refinement and development based on immunological insights gleaned from these experimental investigations. Therefore, the focus is on in-depth characterisation of the elicited immune response to rationally-designed model immunogens that may inform the design process of actual vaccines. This experimental medicine approach is only now possible due to unprecedented progress in our abilities to study the human immune system and to obtain complete information on immune responses to vaccination, since performing research on the human immune system is now almost as easy as it has been in mice. The main focus of this study will be to determine which of the design strategies is able to prime human germline (naive) B cells and drive antibody responses towards induction of neutralising antibody breadth.

Our range of model immunogens will be based on the envelope (Env) glycoprotein of HIV-1, which is the only target of neutralising antibodies, and therefore the only virally-encoded immunogen relevant for induction of such antibodies by immunisation. To ensure reproducibility of results and the highest level of volunteer safety, all immunogens will be manufactured under cGMP, using techniques applied to vaccine immunogens.

Env has extensive amino acid variation, structural and conformational instability, and immunodominance of hypervariable regions (Kwong PD, 2011; Sattentau QJ, 2013). We will design soluble immunogens that closely mimic the native viral trimer *in situ*, but that incorporate design strategies that may alter the intrinsic viral immune evasion mechanisms (Sanders RW, 2013). Env is made up of three identical complexes (trimers) each of which contains two molecules, gp120 and gp41 that can be modified to make a soluble molecule called gp140, upon which our immunogens are based. We have developed model consensus gp140 Env trimers (consensus of all global strains) designed to prime B cell responses to common epitopes represented in all HIV-1 subtypes. We have utilised two design strategies to stabilise these in a native-like conformation: ConM SOSIP and ConS UFO. The ConM SOSIP trimer includes novel mutations that include the incorporation of a disulphide linkage between the gp120 and gp41 ectodomain (making up gp140) which prevents their disassociation into monomer subunits.

The ConS UFO includes a short flexible amino-acid linker to tether the gp120 and gp41 subunits together as an alternative strategy to prevent dissociation of the Env trimer. We wish to test both designs to determine the effect on B cell repertoire. To further stabilize global architecture we will employ an EDC crosslinking approach that has been shown to conserve bNAb epitopes, reduce non-antiviral antibody responses, and enhance overall immunogen stability (Schiffner et al., 2015). Thus, in Part 1 of this study we will test EDC ConM SOSIP and EDC ConS UFO versions in parallel.

A critical adjunct to our consensus-based model design is to use cocktails of mosaic gp140 Env trimers designed to overcome the immunodominance of hypervariable regions of Env and to determine whether they will focus antibody responses towards conserved neutralisation epitopes. While designed using computer algorithms, these mosaics represent authentic Env

structures that are fully functional and native in their conformation. Our novel designs aim to eliminate unwanted immunodominant antibody responses and focus B cells towards highly conserved supersites of vulnerability on Env, with particular emphasis on quaternary bNAb epitopes (Julien, JP, 2013; Kong L, 2013; Lyumkis D, 2013). Like the ConM SOPSIP and ConS UFO trimers described above, the mosaic trimers have disulphide linkage which prevents disassociation of gp120 and gp41 into monomer subunits.

In Groups A–E of this study, we will prime with one of the consensus immunogens (ConM or ConS), and boost with a cocktail of two mosaic immunogens (Mos3.1 and Mos3.2).

In Groups F–I of this study, we will explore the use of three mosaic immunogens (Mos3.1, Mos3.2 and Mos3.3) used sequentially (in a series of different orders), or as a cocktail, to focus B cell responses towards conserved areas of Env. To amplify these responses we intend to give a final boosting immunization with both consensus immunogens (ConM and ConS).

In Groups J–M we will use two HIV-1 envelope sequences (AMC011 and 763 SOSIP) which the EAVI2020 programme has found to be associated with the early induction of broadly neutralising antibodies. The sequences were identified from two HIV infected individuals that displayed HIV neutralisation breadth early in infection (less than 12 months). This is unusual as typically neutralisation breadth is only seen to develop in <5% of subjects and only after a period of more than 2 years. We seek to test the hypothesis that these two envelope sequences may display unique potential to prime and/or drive the induction of broadly neutralising antibody responses in HIV negative healthy volunteers. To test this hypothesis the EAVI2020 consortium has manufactured stabilised versions of these two envelope proteins to GMP. These will be used alone and in various combinations with consensus and mosaic immunogens to determine the extent to which they can drive the induction of broadly neutralising antibodies. Responses to these unique envelope sequences will be compared and contrasted to the consensus and mosaic immunogens evaluated in Groups A–I.

The extent to which these different strategies may induce neutralising breadth, and the identification of the mechanisms and drivers involved, can only be determined empirically through human immunogen challenge studies. The results from these studies will help inform the future direction of additional refinement of HIV vaccine strategies for the induction of protective antibodies.

Objectives

The objective of this experimental medicine study is to determine the extent to which different prime-boost combinations influence serum neutralising antibody breadth and associated B and T cells responses.

We hypothesise that the different prime-boost model immunogen combinations will have differential impact on:

a) the magnitude and breadth of induced serum neutralising antibodies;

and

b) the induced B- and T-cell responses in peripheral blood

We will investigate this by challenging the immune system of healthy adults with various model immunogens based on HIV-1 Env (ConM and ConS, with and without EDC stabilisation; Mos3.1, Mos3.2 and Mos3.3, and AMC011 and 763 SOSIP) in different prime-boost combinations.

In Groups A–I we will investigate the impact of a later immunisation challenge with either consensus or mosaic gp140 Envs to determine any additional effect on breadth of antibody neutralisation.

The primary outcome will be measurement of specific viral neutralisation activity of serum antibodies. Exploratory outcomes will include titres of binding antibodies in serum and the characterisation of blood B and T cell responses.

Data derived from these studies will help to inform how the structure of rationally-designed immunogens drives the immune system towards developing broadly neutralising antibodies that are rarely induced under natural conditions.

Primary Outcomes

- Serum titres of neutralising antibodies to virus expressing ConM and ConS envelopes
- Serum titres of neutralising antibodies to virus expressing Mosaic envelopes (Mos3.1, Mos3.2 and Mos3.3)
- Serum titres of neutralising antibodies to virus expressing AMC011 and 763 SOSIP envelopes

Exploratory Outcomes

- These will include: measurement of binding antibodies in serum; assessment of neutralisation breadth using a wider panel of globally circulating HIV strains; measurement of serum antibody function (ADCC, ADCP, FcR dimer binding); measurement of the magnitude and phenotype of B cell (plasmablast and memory B cells) and T cell responses (CD4 and CD8) in peripheral blood mononuclear cells (PBMC). Some endpoints will include only a subset of enrolled volunteers. Paxgene tubes for blood transcriptomics will be collected only from group A (see Table 2) because it is not expected that responses will differ at this level between groups. Blood transcriptomic profiles induced by immunisation will be correlated with the magnitude of induced serum antibody response.

Fine needle aspiration

- In addition, the feasibility and safety of lymph node aspiration will be explored, and immunogen induced germinal centre B and T cell responses will be assessed, in volunteers from groups B-M willing to attend additional visits to have an ultrasound guided fine needle lymph node aspiration from axillary, cervical or inguinal lymph nodes. Fine needle aspiration biopsies will occur in line with the sub-study protocol in Appendix 1.
- This will be optional and participants will be provided with a separate fine needle aspiration information sheet and consent form. The participants will be reviewed by the medical team for any contraindications to fine needle aspiration prior to the procedure being carried out.
- An additional blood sample of 42 ml of peripheral blood will be collected at each fine needle aspiration visit to compare the T- and B-cell populations by flow cytometry and to compare serum neutralising antibody activity.

Statistics and Sample Size

It is difficult to give an estimate of the power of group comparisons using quantitative antibody titre outcomes at this stage as this is dependent on the number of responders, and the large number of experimental parameters in this experimental study. The sample size is based on the previous studies of HIV-1 gp140 immunogens (Joseph S, 2017, Cosgrove CA, 2016, Kratochvil S, 2017, Lewis DJ, 2011). It is not the remit of this exploratory study to recruit a sufficient number of volunteers to be statistically confident about the differences between groups. By the end of this study, 8–10 volunteers will have been exposed to each schedule and this provides confidence around the response/event proportions of 0–100% in Table 1.

Table 1: Sample Size

Number of "responders"	Proportion if n=10	95% confidence interval[1]
0	0%	0 – 31%
2	20%	3 – 56%
4	40%	12 – 74%
6	60%	26 – 88%
8	80%	44 – 98%
10	100%	69 – 100%
**Number of "responders"**	**Proportion if n=8**	**95% confidence interval[2]**
0	0%	0–32%
1	12.5%	2–47%
2	25%	7–59%
3	37.5%	14–69%
4	50%	22–78%

5	62.5%	31–86%
6	75%	41–93%
8	100%	68–100%

[1, 2] Clopper-Pearson or Wilson method (suitable for small sample sizes)

Number (percent) of responders, and median [IQR] and mean (range) of titre responses will be tabulated by group. Group comparisons of titre levels will be conducted via Kruskal-Wallis test or Wilson method suitable for small sample sizes.

Volunteers & Withdrawal Criteria

We will recruit 114 healthy male and females adults into five groups of 10 each (A-E) and eight groups of 8 each (F–M), see Table 2. Recruitment will select potential volunteers who are willing to undergo serial blood draws. Health status will be determined at screening which will comprise a medical history, symptom-directed physical examination, vital signs, urine pregnancy test (women only), HIV screen, and routine safety blood draws (full blood count; renal, liver and bone panels).

Additional volunteers may be enrolled to replace early withdrawals. The reason(s) for withdrawal will be recorded in volunteer's files.

All volunteers are free to withdraw from the study at any time, for any reason, without affecting their future medical care. The PI may decide to withdraw a volunteer if the investigator deems that continuing might compromise the volunteer's wellbeing or interfere with the achievement of the study's objectives.

Table 2: Schedule of immunisations and doses of model immunogens

Group	N	Months			
		0	3	6	12
		Part 1			Part 2
A	10	ConM SOSIP<br>100 μg	ConM SOSIP<br>100 μg	ConM SOSIP<br>100 μg	Mos3.1 + Mos3.2<br>100ug (2x50ug)
B	10	EDC ConM SOSIP<br>100 μg	EDC ConM SOSIP<br>100 μg	EDC ConM SOSIP<br>100 μg	Mos3.1 + Mos3.2<br>100ug (2x50ug)
C	10	ConS UFO<br>100 μg	ConS UFO<br>100 μg	ConS UFO<br>100 μg	Mos3.1 + Mos3.2<br>100ug (2x50ug)
D	10	EDC ConS UFO<br>100 μg	EDC ConS UFO<br>100 μg	EDC ConS UFO<br>100 μg	Mos3.1 + Mos3.2<br>100ug (2x50ug)
E	10	ConS UFO<br>100 μg	ConS UFO<br>100 μg	ConM SOSIP<br>100 μg	Mos3.1 + Mos3.2<br>100ug (2x50ug)
Group	N	Months			
		0	2	4	6
		Part 1			Part 2
F	8	Mos3.1<br>100 μg	Mos3.2<br>100 μg	Mos3.3<br>100 μg	ConM SOSIP +<br>ConS UFO<br>50 μg + 50 μg
G	8	Mos3.2<br>100 μg	Mos3.1<br>100 μg	Mos3.3<br>100 μg	ConM SOSIP +<br>ConS UFO<br>50 μg + 50 μg
H	8	Mos3.3<br>100 μg	Mos3.2<br>100 μg	Mos3.1<br>100 μg	ConM SOSIP +<br>ConS UFO<br>50 μg + 50 μg
I	8	Mos3.1<br>33 μg<br>Mos3.2<br>33 μg<br>Mos3.3<br>33 μg	Mos3.1<br>33 μg<br>Mos3.2<br>33 μg<br>Mos3.3<br>33 μg	Mos3.1<br>33 μg<br>Mos3.2<br>33 μg<br>Mos3.3<br>33 μg	ConM SOSIP +<br>ConS UFO<br>50 μg + 50 μg

Note: Participant 060, in Group F, received (in error) Mos3.3 100 μg at Month 2. She may continue to be challenged if she receives Mos3.2 100 μg at Month 4, and is challenged at Month 6 per the table above.

Table continues overleaf**Table 2 (continued): Schedule of immunisations and doses of model immunogens**

Group	N	Month		
		0	2	4
J	8	763 SOSIP<br>100 µg	763 SOSIP<br>100 µg	763 SOSIP<br>100 µg
K	8	AMC011<br>100 µg	AMC011<br>100 µg	AMC011<br>100 µg
L	8	763 SOSIP<br>50 µg<br>+<br>AMC011 SOSIP<br>50 µg	763 SOSIP<br>50 µg<br>+<br>AMC011 SOSIP<br>50 µg	763 SOSIP<br>50 µg<br>+<br>AMC011 SOSIP<br>50 µg
M	8	763 SOSIP<br>AMC011<br>ConM SOSIP<br>Mos3.1<br>Mos3.2<br>(each at 20 µg)	763 SOSIP<br>AMC011<br>ConM SOSIP<br>Mos3.1<br>Mos3.2<br>(each at 20 µg)	763 SOSIP<br>AMC011<br>ConM SOSIP<br>Mos3.1<br>Mos3.2<br>(each at 20 µg)

<u>Inclusion Criteria</u>

1. Healthy male and female volunteers aged between 18 and 55 years.
2. Available for **<u>ALL</u>** follow-up visits for the duration of the study.
3. Entered and clearance obtained from The Over volunteering Prevention System (TOPS) database (to avoid impact of any co-administered investigational products or treatments on our outcomes).
4. Women of childbearing[i] potential willing to use a highly effective method of contraception[ii] for the duration of the study until a minimum of 12 weeks after the final injection. Periodic abstinence (calendar, symptothermal and post-ovulation methods) and withdrawal are not acceptable methods of contraception.
5. Willing and able to give written informed consent.

i A woman will be considered of childbearing potential following menarche and until becoming post-menopausal (no menses for 12 months without an alternative medical cause) unless permanently sterile. Permanent sterilisation methods include hysterectomy, bilateral salpingectomy and bilateral oophorectomy. A post-menopausal state is defined as no menses for 12 months without an alternative medical cause.

ii The following methods are considered highly effective:
- combined (estrogen and progestogen containing) hormonal contraception associated with inhibition of ovulation – oral, intravaginal or transdermal;
- progestogen-only hormonal contraception associated with inhibition of ovulation – oral, injectable or implantable
- intrauterine device (IUD);
- intrauterine hormone-releasing system (IUS);
- bilateral tubal occlusion;
- vasectomised partner, where the vasectomised partner has received medical assessment of the surgical success; and
- sexual abstinence, defined as refraining from heterosexual intercourse – must be the preferred and usual lifestyle of the participant.

Exclusion Criteria

1. History of any medical, psychological or other condition, clinically significant laboratory result at screening, or use of any medications which, in the opinion of the investigators, would interfere with the study objectives or volunteers safety.
2. Any history of angioedema
3. History of urticaria deemed significant by the Chief Investigator
4. HIV-1 or HIV-2 antibody positive or indeterminate upon screening, or history of receipt of Env-based HIV immunogens (which would render the volunteers non-naive to the model immunogens).
5. Unable to read and/or speak English to a fluency level adequate for the full comprehension of study procedures and consent.

Recruitment & Randomisation

This is a single-blind study with volunteers being randomised into five groups of n=10 (Groups A-E) and eight groups of n=8 (Groups F–M), stratified by gender. Volunteers will be blinded to their treatment regimes, and laboratory teams undertaking immunological analysis will be blinded to group and dosing regimen to prevent in-house analysis bias. The clinical team will remain un-blinded throughout.

Volunteers will be recruited from the general population in London and the surrounding areas by means of paper and electronic advertising. We will also use a Healthy Volunteer database held at the Imperial Clinical Research Facility. We will register volunteers as patients of Imperial College Healthcare NHS Trust before they are consented into the study. Volunteers will also be informed at screening and during the course of the experimental study protocol that the immune challenges will not provide protection against HIV acquisition, and that normal HIV prevention strategies should be maintained.

Once all inclusion/exclusion criteria have been met, eligible volunteers will be enrolled into the study and randomly assigned into one of the nine treatment groups. A study ID will be generated once randomisation has been completed.

Consent

Signed written consent to enter the study must be obtained from each volunteer only after a full explanation has been given, an information leaflet provided, and at least 24-48 hours allowed for consideration. The right of the volunteer to refuse to participate and to withdraw consent at any time from the protocol without giving reasons will be respected.

Model Immunogens

Model immunogens will be obtained from Polymun Scientific, Austria, and stored in temperature controlled conditions, on-site at the Imperial CRF before administration. To ensure reproducibility of results and the highest level of volunteer safety, all immunogens will be manufactured under cGMP, using techniques and specifications applied to vaccines. Recombinant HIV-1 trimeric gp140 Env proteins: ConM SOSIP gp140, ConS UFO gp140, and chemically fixed versions: EDC-ConM SOSIP and EDC-ConS UFO; the mosaic immunogens: Mos3.1, Mos3.2 and Mos3.3; and 763 SOSIP and AMC011, manufactured to EU cGMP quality standards by Polymun Scientific GmbH (Austria) will be mixed with adjuvant: MPLA liposomes manufactured to EU GMP quality standards by Polymun Scientific. All model immunogens will be used at the dosage of 100 μg and will be admixed with 500 μg MPLA formulated in liposomes and administered by intramuscular injection into the left or right deltoid muscle.

Immunogens, adjuvant and dose justification

Env glycoprotein and adjuvant vaccines have been administered without concerning adverse effect in numerous studies worldwide over many years. Extensive safety data for the use of monomeric recombinant gp120 HIV-1 Env glycoprotein vaccines include the large Phase III Vaxgen and RV144 trials - albeit formulated with different adjuvants (Andersson et al. 2011; Su et al. 2000; UNIADS Report 2013). In the RV144 trial, the alum adjuvanted gp120 protein (AIDSVAX B/E) was injected along with ALVAC (canarypox expressing *gag pro* and *Env* genes) after two priming immunisations with ALVAC alone. There were no safety concerns in that trial of over 16,000 healthy volunteers in Thailand.

Previous work by our own group, and others internationally, have used recombinant native HIV-1 Env glycoprotein gp140 in a number of CTIMPs, administered intramuscularly (I.M.) at the dose of 100μg adjuvanted with GLA-AF, an alternative MPLA (non-liposomal) formulation, as well as with other adjuvants such as alum and MF59. The dose of model gp140 immunogens we propose to use in this protocol is therefore identical to the dose of native gp140 (100μg) used in three previous UK clinical trials: MUCOVAC2 (EudraCT 2010-019103-27; Cosgrove CA, 2016); UKHVC Spoke 003 (EudraCT 2012-003277-26; Joseph S, 2017); X001 (EudraCT 2013-001032-22; Kratochvil S, 2017). An intradermal dose of 50ug was acceptable when used in CUTHIVAC002 (EudraCT 2015-001023-23; Cheeseman HM, 2018). The proposed dose of native gp140 was safe, and volunteers made good binding antibody responses to the recombinant gp140, although very limited levels of neutralising antibodies. These previous

studies were performed with a single recombinant HIV-1 clade C trimeric gp140 Env glycoprotein derived from a Chinese viral isolate 97CN54. Additional studies have been performed with gp140 proteins at this dose (100 µg). A South African subtype C strain TV1 trimeric gp140 protein (100 µg) combined with MF59 adjuvant was safely administered to 114 volunteers primed with DNA/MVA (Churchyard G, 2016); a purified trimeric gp140 Env derived from the subtype B strain SF162 (100 µg) in MF59 was administered to 80 subjects with or without DNA priming (Spearman P, 2011); and a subtype D strain UG92005 trimeric gp140 Env (100 µg) combined with 500 µg alum was given twice to 9 volunteers (Hurwitz JL, 2008).

To adjuvant the protein this study will use MPLA in liposomal suspension. MPLA has been widely used in liposomal adjuvants for over 20 years (Alving CR, 2011). When given intramuscularly no systemic toxicity has been associated with doses up to 2200 µg (Fries LF 1992, Heppner DG, 1996, Alving CR, 2011). We have selected a dose of 500 µg as optimal based on non-human primate immunogenicity studies of gp140, in which there was no toxicity.

We also commissioned GLP-compliant preclinical toxicology testing in rabbits (RTC STUDY NO. A3063, Research Toxicology Centre S.p.A., Italy) to mimic the human challenge studies, in which repeated injections of ConM SOSIP, EDC-ConM SOSIP, ConS UFO and EDC-ConS UFO with MPLA, and of ConM SOSIP and MPLA alone, were administered into the lateral surface of the quadriceps muscle of both legs. No relevant clinical signs were observed, and no reaction at injection sites was observed in any animal treated with the test item during the study. In this model the challenge agents and schedule did not induce adverse effects at systemic or local levels.

Reporting Procedures

Any adverse events will be solicited at each attendance, reviewed by clinicians and recorded. In addition, participants will be asked to complete a symptom diary on the evening after each immunogen challenge, and every evening for 6 days thereafter, and entries will be reviewed by clinicians when they visit the investigator site. Any serious adverse events should be reported to the Sponsor and Research Ethics Committee where in the opinion of the Chief Investigator, the event was: 'related', i.e. resulted from the administration of any of the research procedures; and 'unexpected', i.e. an event that is not listed in the protocol as an expected occurrence. Reports of related and unexpected SAEs should be submitted within 15 days of the Chief Investigator becoming aware of the event, using the NRES SAE form for non-IMP studies.

Possible Adverse Reactions and Adverse Events monitoring

As immediate anaphylaxis may rarely be associated with immunogen challenge (less than 1 in 1 million doses) volunteers will remain in the investigation unit for at least one hour after

immunogen challenge. The symptom diary completed by participants after each immunogen challenge will support the completeness and accuracy of adverse events recording at each attendance.

No serious adverse reactions are anticipated given the wide use of recombinant HIV-1 Env proteins in previous studies, and safe record of using MPLA as a common adjuvant. Various local, non-serious adverse reactions may be anticipated after immunogen challenge, expected to be of short duration, resolving within 1-7 days. These include pain, tenderness, erythema/discolouration, warmth, itching and swelling at the site of injection. Less frequent systemic non-serious adverse reactions associated with immunogen challenge may also occur and include: raised temperature, chills/rigors, myalgia/flu-like general muscle aches, malaise, excess fatigue, headache, nausea/vomiting; and, very rarely abdominal pain, diarrhoea and/or sore throat. These are also anticipated to resolve within a few days (<7 days).

Adverse events associated with lymph node sampling are described in the sub-study protocol, in Appendix 1.

It will be made clear to volunteers that they will be free to withdraw from the study at any time without providing a reason. Any significant, severe adverse reactions will lead to withdrawal of the individual and any serious adverse reactions will terminate the whole study.

Volunteers will be provided with 24 hour emergency contact numbers and clear instructions to call if they feel unwell following immunogen challenge.

Schedule of visits, immunogen challenges, procedures and samples

Volunteers will be screened, have their first immunogen challenge and will be followed for 7 months (Groups J-M), 9 months (Groups F–I) or 13 months (Groups A–E) thereafter. It is expected to take approximately 12 months to enrol 114 volunteers.

Volunteers will be enrolled in the groups described in Table 2 and blood sampling will be carried out at specific time points during the study (see Table 3).

Avoidance of pregnancy during the study

Women of childbearing potential are recommended to use highly effective contraception for the duration of the study until a minimum of 12 weeks after the second injection. The immunogen challenge contains 500 µg of MPLA, which is similar to the 50 µg of 3-O-desacyl-4'-monophosphoryl lipid A in the licensed vaccine against Hepatitis B, Fendrix (GSK). The summary of medicinal product characteristics (SmPC) for this product states that although there are no data from the use of Fendrix in pregnant women, animal studies do not indicate direct or indirect harmful effects with respect to pregnancy, embryonal/foetal development, parturition or postnatal development. (https://www.medicines.org.uk/emc/product/137/smpc). The authorised product Cervarix (GSK) also contains MPLA. During clinical development 5,387 women who had received

Cervarix reported pregnancy. There was no evidence of increased risk to the pregnancy in these women (https://www.medicines.org.uk/emc/product/1177/smpc#gref).

Study Management

The day-to-day management of the study will be co-ordinated through Dr Katrina Pollock, the Chief Investigator, and the study team at the Hammersmith Hospital (clinical) and Imperial College London's St Mary's Campus (laboratory). Study management decisions will be discussed with the wider clinical team, and authorised by the Chief Investigator and Scientific Co-Investigator.

A Data Safety Monitoring Committee will be formed and will meet to review the accumulated safety data after approximately half of the total number of immunogen challenges have been administered. The DSMC will be asked to make a recommendation such as: continuation of the study with no proposed changes to the study protocol; or early termination due to adverse reactions; or amendment of the study protocol. After the initial review meeting the DMSC will be sent periodic safety reports, approximately every 3 months, and all SAR reports. Meetings will be scheduled *ad hoc*, at the request of the CI or DMSC members. Further details are in the DSMC Charter.

Reimbursement of Volunteers

Volunteers will be paid for their time and effort to cover their travel expenses to the study site and any inconvenience caused due to study participation. Volunteers will receive £100 per completed study visit, and a £100 bonus if they attend all study visits on time and comply with all study requirements, as a lump sum at the end of their participation.

Table 3: Schedule of Study Events – Groups A–E

		Part 1																Part 2				
**Visit Number**		**1**	**2**	**3**	**4**	**5**	**6**	**7**	**8**	**9**	**10**	**11**	**12**	**13**	**14**	**15**	**16**	**17**		**19**	**20**	**21**
**Visit Month**		**M0**					**M3**					**M6**						**M12**				
**Visit Week**		**0**	**0**	**1**	**2**	**4**	**12**	**12**	**13**	**14**	**16**	**24**	**24**	**25**	**26**	**28**	**36**	**48**		**49**	**50**	**52**
**Visit Day**		**0**	**1**	**7**	**14**	**28**	**84**	**85**	**91**	**98**	**112**	**168**	**169**	**175**	**182**	**196**	**252**	**336**		**343**	**350**	**364**
**Visit Windows (Days)**	**Screening**	**0**	**0**	**± 1**	**± 2**	**± 2**	**-7/+14**	**0**	**± 1**	**± 2**	**± 2**	**-7/+14**	**0**	**± 1**	**± 1**	**± 2**	**± 2**	**-7/+14**		**± 1**	**± 2**	**± 2**
Immunogen challenge[(1)]		X					X					X						X				
Informed consent	X																					
Medical history/ demographics / height and weight	X																					
Symptom-directed physical examination[(2)]	X	(X)	(X)	(X)	(X)	(X)	(X)	(X)	(X)	(X)	(X)	(X)	(X)	(X)	(X)	(X)	(X)	(X)		(X)	(X)	X
Vital Signs[(3)]	X	X		X			X		X			X		X				X		X		
Full blood count, serum renal, liver, bone panels & HIV-1 & 2 antibodies[7]	X																					X[7]
Provide symptom diary		X					X					X						X				
Review symptom diary			X	X				X	X				X	X						X		
Urine Pregnancy Test (Women)	X	X					X					X						X				
Blood for Serum – *Immunogenicity*		X		X	X	X	X		X	X	X	X		X	X	X	X	X		X	X	X
Blood for PBMC – *Immunogenicity* (*Group A only)		X	X*	X			X	X*	X			X	X*	X	X	X	X	X		X	X	X
Blood RNA (Paxgene) (*Group A only)		X*	X*	X*			X*	X*	X*			X*	X*	X*								
Lymph Node Aspirate (**Group B – E only)		X**,4			X**,5					X**,6												

[1] The second, third and fourth immunogen challenges (visits 6, 11 and 17) can be shifted by -7 or up to +14 days. Subsequent visits will be shifted by the same number of days to ensure the interval to subsequent visits are maintained

[2]Symptom-directed physical examination maybe preformed at the discretion of the Chief Investigator/delegate

[3]At screening and before immunogen challenge, and at 60 (+/-15) min and 7 (+/-1) days post-challenge

[4] FNA sample window 1: from enrolment to Day 0 (prior to first immunogen challenge).

[5]FNA sample window 2: Day 7 to Day 84. Aim for FNA Samples to be obtained at Day 14 (2 weeks after first immunogen challenge)

[6]FNA sample window 3: Day 85 to Day 364 (1 to 280 days post second challenge). Aim for FNA samples to be obtained at Day 98 (2 weeks after second immunogen challenge). If a participant has two sampling occasions during this window, the samplings should be at least 7 days apart.

[7]HIV antibodies at screening only

*Group A only

**Group B-E only

Table 3 continued: Schedule of Study Events – Groups F–I

		Part 1												Part 2				
**Visit Number**		**1**	**2**	**3**	**4**	**5**	**6**	**7**	**8**	**9**	**10**	**11**	**12**	**13**	**14**	**15**	**16**	**17**
**Visit Month**		**M0**				**M2**				**M4**				**M6**				**M9**
**Visit Week**		**0**	**1**	**2**	**4**	**8**	**9**	**10**	**12**	**16**	**17**	**18**	**20**	**24**	**25**	**26**	**28**	**36**
**Visit Day**		**0**	**7**	**14**	**28**	**56**	**63**	**70**	**84**	**112**	**119**	**126**	**140**	**168**	**175**	**182**	**196**	**252**
**Visit Windows (Days)**	**Screening**	**0**	**± 1**	**± 2**	**± 2**	**-7/+14**	**± 1**	**± 2**	**± 2**	**-7/+14**	**± 1**	**± 2**	**± 2**	**-7/+14**	**± 1**	**± 2**	**± 2**	**-7/+14**
Immunogen challenge[(1)]		X				X				X				X				
Informed consent	X																	
Medical history/ demographics / height and weight	X																	
Symptom-directed physical examination[(2)]	X	(X)	(X)	(X)	(X)	(X)	(X)	(X)	(X)	(X)	(X)	(X)	(X)	(X)	(X)	(X)	(X)	X
Vital Signs[(3)]	X	X	X			X	X			X	X			X	X			
Full blood count, serum renal, liver, bone panels & HIV-1 & 2 antibodies[4]	X																	X[4]
Provide symptom diary		X				X				X				X				
Review symptom diary			X				X				X				X			
Urine Pregnancy Test (Women)	X	X				X				X				X				
Blood for Serum – *Immunogenicity*		X		X	X	X		X	X	X	X	X	X	X	X	X	X	X
Blood for PBMC – *Immunogenicity*		X	X			X	X			X	X	X	X	X	X	X	X	X
Lymph Node Aspirate		X*		X**				X***										

1 The second, third and fourth immunogen challenges (Visits 5, 9 and 13) can be shifted up to -7 to +14 days. Subsequent visits will be shifted by the same number of days to ensure the interval to subsequent visits are maintained

2 Symptom-directed physical examination maybe preformed at the discretion of the Chief Investigator/delegate

3 At screening and before immunogen challenge, and at 60 (+/-15) min and 7 (+/-1) days post-challenge

4 HIV antibodies at screening only

* FNA sample window 1: from enrolment to Day 0 (prior to first immunogen challenge).

** FNA sample window 2: Day 7 to Day 56. Aim for FNA Samples to be obtained at Day 14 (2 weeks after first immunogen challenge)

***FNA sample window 3: Day 57 to Day 252 (1 to 196 days post challenge). Aim for FNA samples to be obtained at Day 70 (2 weeks after second immunogen challenge). If a participant has two sampling occasions during this window, the samplings should be at least 7 days apart.

Table 3 continued: Schedule of Study Events – Groups J–M

Visit Number		1	2	3	4	5	6	7	8	9	10	11	12	13
Visit Month		M0				M2				M4				M7
Visit Week		0	1	2	4	8	9	10	12	16	17	18	20	28
Visit Day		0	7	14	28	56	63	70	84	112	119	126	140	196
Visit Windows (Days)	Screening	0	± 1	± 2	± 2	-7/+14	± 1	± 2	± 2	-7/+14	± 1	± 2	± 2	-7/+14
Immunogen challenge[(1)]		X				X				X				
Informed consent	X													
Medical history/ demographics / height and weight	X													
Symptom-directed physical examination[(2)]	X	(X)	(X)	(X)	(X)	(X)	(X)	(X)	(X)	(X)	(X)	(X)	(X)	X
Vital Signs[(3)]	X	X	X			X	X			X	X			
Full blood count, serum renal, liver, bone panels & HIV-1 & 2 antibodies[4]	X													X[4]
Provide symptom diary		X				X				X				
Review symptom diary			X				X				X			
Urine Pregnancy Test (Women)	X	X				X				X				
Blood for Serum – *Immunogenicity*		X		X	X	X		X	X	X	X	X	X	X
Blood for PBMC – *Immunogenicity*		X	X			X	X			X	X	X	X	X
Lymph Node Aspirate		X*		X**				X***						

1 The second and third immunogen challenges (Visits 5 and 9) can be shifted up to -7 to +14 days. Subsequent visits will be shifted by the same number of days to ensure the interval to subsequent visits are maintained

2 Symptom-directed physical examination maybe preformed at the discretion of the Chief Investigator/delegate

3 At screening and before immunogen challenge, and at 60 (+/-15) min and 7 (+/-1) days post-challenge

4 HIV antibodies at screening only

* FNA sample window 1: from enrolment to Day 0 (prior to first immunogen challenge).

** FNA sample window 2: Day 7 to Day 56. Aim for FNA Samples to be obtained at Day 14 (2 weeks after first immunogen challenge)

***FNA sample window 3: Day 57 to Day 196 (1 to 140 days post challenge). Aim for FNA samples to be obtained at Day 70 (2 weeks after second immunogen challenge). If a participant has two sampling occasions during this window, the samplings should be at least 7 days apart.

Transfer, sample storage and analysis

All PBMC and Serum samples (for immunological analysis) obtained at the Imperial CRF will be transferred to the Shattock Laboratory at Imperial College London, St Mary's Hospital campus, for analysis. Lymph node samples will also be transferred to the Shattock Laboratory.

Samples of cryopreserved PBMC, lymph node and serum will be stored and analysed internally as per the Shattock Laboratory working instructions for up to 10 years following study completion. As part of our consortium (EAVI2020), our collaborators outside of the UK might also undertake further immunological analysis of data and / or samples generated from the study. These samples will be pseudoanonymised so that our study collaborators are blinded to the volunteer's personal identifiable information.

Restarting participation following a pause due to COVID-19 or other reason

Where participation of enrolled subjects was paused due to the COVID-19 pandemic, or other reason, we will restart affected participants at their next scheduled immunogen challenge visit. In order to check that their health status hasn't changed significantly since we last saw them, they will attend the Imperial CRF for routine safety blood tests (full blood count; renal, liver and bone panels) before the challenge visit. The need for this additional visit is at the investigator's discretion; it might not be required for participants who were paused for a relatively short time.

For some affected participants, their most recent visit before restarting was an immunogen challenge visit at which follow-up assessments were performed but no immunogen was injected. These participants will repeat this visit, **with** immunogen challenge.

Participants will be paid for extra visits.

Concomitant medication

We will record relevant concomitant medication taken by participants, including vaccines, in order to assess their potential impact on the study's outcomes. Participants will be encouraged to have an authorised COVID-19 vaccine if they are offered one, but we will request that they have it at least 14 days before or after immunogen challenge in the study, and at least 28 days will be encouraged. For seasonal influenza vaccine we will request that they have it at least 7 days before or after immunogen challenge in the study, and at least 28 days will be encouraged. Participants will be encouraged to receive NHS vaccines in the opposite arm from the immunogen challenge.

Ethics Approval

The Chief Investigator will obtain approval from the HRA and Research Ethics Committee. The study must also receive confirmation of capacity and capability from Imperial College Healthcare NHS Trust. The study will be conducted in accordance with the recommendations for physicians involved in research on human subjects adopted by the 18th World Medical Assembly, Helsinki 1964 and later revisions, and with ICH GCP.

Confidentiality

The Chief Investigator will preserve the confidentiality of volunteers taking part in the study. Readily identifiable volunteer data will be held securely at the NHS site hosting the study. All volunteer samples and data leaving the NHS site will be identified only by a unique study code,

and hence will be link-anonymised. All data (results generated from the study) will be anonymised and stored on Imperial College London servers for 10 years.

Indemnity

Imperial College London holds negligent harm and non-negligent harm insurance policies, which apply to this study. For harm caused by staff with substantive NHS employment contracts, the NHS indemnity scheme or professional indemnity will apply.

Sponsor

Imperial College London will act as the main Sponsor for this study. Delegated responsibilities will be assigned to Imperial College Healthcare NHS Trust as necessary.

Finance & Funding

This study is funded by a grant from the European Commission in the H2020 Framework Programme. The investigators do not receive any payment for this study.

Monitoring, Audits, Inspection & End of Study Definition

The study may be subject to audit by Imperial College London under their remit as sponsor, the Study Coordination centre and other regulatory bodies to ensure adherence to GCP. On-site monitoring will be delegated to a trained monitor to ensure that the study is conducted in compliance with human subjects' protection and other research regulations and guidelines, recorded and reported in accordance with the protocol, is consistent with SOPs, GCP and locally accepted practices. The investigators, as well as volunteers through consenting to the study, agree that the monitor may inspect study facilities and source records (e.g., informed consent forms, clinic and laboratory records, other source documents), as well as observe the performance of study procedures. Such information will be treated as strictly confidential and will under no circumstances be made publicly available. The study will be closed when all volunteers have made their final visit.

Storage of research data and archiving

All data will be archived in accordance with the Imperial College London archiving policy implemented by the sponsor, Imperial College London. The archive facility is:
ACRU Store
Charing Cross Hospital
London
W6 8RP

Publication Policy

Results from this study will be disseminated locally and to the wider scientific community at conferences and in published open-access peer-reviewed journals, as appropriate. Any data used for publication will be fully anonymised. We plan to publish the data and hold onto the data for 10 years.

References

Alving CR, Rao M, Steers NJ, Matyas GR, Mayorov AV. (2012) "Liposomes containing lipid A: an effective, safe, generic adjuvant system for synthetic vaccines." Expert Rev Vaccines. 11(6):733-44.

Andersson KM, Paltei AD, and Owens DK. (2011) "The Potential impact of an HIV vaccine with rapidly waning protection on the epidemic in Southern Africa: examining the RV144 trial results." Vaccine 18; 29(36):6107-6112.

Churchyard G, Mlisana K, Karuna S, Williamson AL, Williamson C, Morris L, Tomaras GD, De Rosa SC, Gilbert PB, Gu N, Yu C, Mkhize NN, Hermanus T, Allen M, Pensiero M, Barnett SW, Gray G, Bekker LG, Montefiori DC, Kublin J, Corey L. (2016) "Sequential Immunization with gp140 Boosts Immune Responses Primed by Modified Vaccinia Ankara or DNA in HIV-Uninfected South African Participants." PLoS One. 1;11(9):e0161753.

Cheeseman HM, Day S, McFarlane L, Fleck S, Miller A, Cole T, Sousa-Santos N, Cope AV, Cizmeci D, Tolazzi M, Hwekwete E, Hannaman D, Kratochvil S, McKay P, Chung A, Kent S, Cook A, Scarlatti G, Abraham S, Combadiere B, McCormack S, Lewis D, Shattock R. (2018) "Combined skin and muscle DNA priming provides enhanced humoral responses to an HIV-1 clade C envelope vaccine" Hum Gene Ther. doi.org/10.1089/hum.2018.075.

Cosgrove CA, Lacey CJ, Cope AV, Bartolf A, Morris G, Yan C, Baden S, Cole T, Carter D, Brodnicki E, Shen X, Joseph S, DeRosa SC, Peng L, Yu X, Ferrari G, Seaman M, Montefiori DC, Frahm N, Tomaras GD, Stöhr W, McCormack S, Shattock RJ. (2016) "Comparative Immunogenicity of HIV-1 gp140 Vaccine Delivered by Parenteral, and Mucosal Routes in Female Volunteers; MUCOVAC2, A Randomized Two Centre Study." PLoS One. 9; 11(5):e0152038.

Fries LF, Gordon DM, Richards RL, Egan JE, Hollingdale MR, Gross M, Silverman C, Alving CR. (1992) "Liposomal malaria vaccine in humans: a safe and potent adjuvant strategy." Proc Natl Acad Sci U S A. 1; 89(1):358-62.

Heppner DG, Gordon DM, Gross M, Wellde B, Leitner W, Krzych U, Schneider I,Wirtz RA, Richards RL, Trofa A, Hall T, Sadoff JC, Boerger P, Alving CR,Sylvester DR, Porter TG, Ballou WR. (1996) "Safety, immunogenicity, and efficacy of Plasmodium falciparum repeatless circumsporozoite protein vaccine encapsulated in liposomes." Journal of Infect Dis. 174(2):361-6.

Hurwitz JL, Lockey TD, Jones B, Freiden P, Sealy R, Coleman J, Howlett N, Branum K, Slobod KS. (2008) "First phase I clinical trial of an HIV-1 subtype D gp140 envelope protein vaccine: immune activity induced in all study participants." AIDS 2;22(1):149-51.

Joseph S, Quinn K, Greenwood A, Cope AV, McKay PF, Hayes PJ, Kopycinski JT, Gilmour J, Miller AN, Geldmacher C, Nadai Y, Ahmed MI, Montefiori DC, Dally L, Bouliotis G, Lewis DJ, Tatoud R, Wagner R, Esteban M, Shattock RJ, McCormack S, Weber J. (2017) "A Comparative Phase I Study of Combination, Homologous Subtype-C DNA, MVA, and Env gp140 Protein/Adjuvant HIV Vaccines in Two Immunization Regimes." Front Immunol 22;8:149.

Julien JP, Cupo A, Sok D, Stanfield RL, Lyumkis D, Deller MC, Klasse PJ, Burton DR, Sanders RW, Moore JP, Ward AB, Wilson IA. (2013) "Crystal structure of a soluble cleaved HIV-1 envelope trimer." Science. 20; 342(6165):1477-83.

Kong L, Lee JH, Doores KJ, Murin CD, Julien JP, McBride R, Liu Y, Marozsan A, Cupo A, Klasse PJ, Hoffenberg S, Caulfield M, King CR, Hua Y, Le KM, Khayat R, Deller MC, Clayton T, Tien H, Feizi T, Sanders RW, Paulson JC, Moore JP, Stanfield RL, Burton DR, Ward AB, Wilson IA. (2013) "Supersite of immune vulnerability on the glycosylated face of HIV-1 envelope glycoprotein gp120." Nat Struct Mol Biol. 20(7):796-803

Kratochvil S, McKay PF, Chung AW, Kent SJ, Gilmore J, Shattock RJ. (2017) "Immunoglobulin G1 Allotype Influences Antibody Subclass Distribution in Response to HIV gp140 Vaccination." Front Immunol. 20; 8:1883.

Kwong PD, Mascola JR, Nabel GJ. (2011) "Rational design of vaccines to elicit broadly neutralizing antibodies to HIV-1." Cold Spring Harb Perspect Med. 1(1):a007278.

Lewis DJ, Fraser CA, Mahmoud AN, Wiggins RC, Woodrow M, Cope A, Cai C, Giemza R, Jeffs SA, Manoussaka M, Cole T, Cranage MP, Shattock RJ, Lacey CJ. (2011) "Phase I randomised clinical trial of an HIV-1(CN54), clade C, trimeric envelope vaccine candidate delivered vaginally." PLoS One. 6(9):e25165.

Lyumkis D, Julien JP, de Val N, Cupo A, Potter CS, Klasse PJ, Burton DR, Sanders RW, Moore JP, Carragher B, Wilson IA, Ward AB. (2013) "Cryo-EM structure of a fully glycosylated soluble cleaved HIV-1 envelope trimer." Science. 20; 342(6165):1484-90.

Mascola JR, and Haynes BF, (2013) "HIV-1 neutralizing antibodies: understanding nature's pathways." Immunol Rev. 254(1):225-44.

Nkolola JP, Bricault CA, Cheung A, Shields J, Perry J, Kovacs JM, Giorgi E, van Winsen M, Apetri A, Brinkman-van der Linden EC, Chen B, Korber B, Seaman MS, Barouch DH. (2014) "Characterization and immunogenicity of a novel mosaic M HIV-1 gp140 trimer." Journal of Virol. 1;88(17):9538-52.

Sanders RW, (2013) "HIV takes double hit before entry." BMC Biol. 7; 10:99.

Sattentau QJ, (2013) "Envelope Glycoprotein Trimers as HIV-1 Vaccine Immunogens." Vaccines. 1(4):497-512.

Spearman P, Lally MA, Elizaga M, Montefiori D, Tomaras GD, McElrath MJ, Hural J, De Rosa SC, Sato A, Huang Y, Frey SE, Sato P, Donnelly J, Barnett S, Corey LJ; HIV Vaccine Trials Network of NIAID. (2011) "A trimeric, V2-deleted HIV-1 envelope glycoprotein vaccine elicits potent neutralizing antibodies but limited breadth of neutralization in human volunteers." Journal of Infect Dis. 15;203(8):1165-73

Su L, Graf M, Zhan Y, von Briesen H, Xing H, Köstler J, Melz, H, Wolf H, Shao Y, and Wagner, R. (2000) "Characterization of a virtually full-length human immunodeficiency virus type 1 genome of a prevalent intersubtype (C/B') recombinant strain in China" J Virol 74(23):11367-11376

Appendix 1 Sub-study Protocol
