# Supplementary material for: Experimental medicine study with stabilised native-like HIV-1 Env immunogens drives long-term antibody responses, but lacks neutralising breadth
Source: eBioMedicine. 2025 Jan 2;112:105544. doi: 10.1016/j.ebiom.2024.105544 (PMC11753977; doi:10.1016/j.ebiom.2024.105544)
Supplement: Supplementary Tables S1-S7 [file mmc5.docx]

**Supplementary Tables**

Table S1 Demographics of the Participants of EAVI_A

Table S2 Inclusion and Exclusion Criteria

Table S3 Summary of the Median antigen-specific IgG memory B cells

Table S4 Summary of neutralisation data from the wider Global panel of HIV viruses.

Table S5 Data from positive broadly neutralising antibody controls against ConM and ConS viruses.

**

Table S6 Alignment of the ConM and ConS Env immunogens

ConM MDRAKLLLLLLLLLLPQAQA-ENLWVTVYYGVPVWKDAETTLFCASDAKAYDTEKRNVWA

ConS MDRAKLLLLLLLLLLPQAQAVENLWVTVYYGVPVWKDAETTLFCASDAKAYDTEVRNVWA

-Signal peptide----||--------------------------------- -----

ConM THCCVPTDPNPQEIVLENVTENFNMWKNNMVEQMHTDIISLWDQSLKPCVKLTPLCVTLN

ConS THACVPTDPNPQEIVLENVTENFNMWKNNMVEQMHTDIISLWDQSLKPCVKLTPLCVTLN

-- --------C1-domain---------------------------||V1-domain--

ConM CTDVNATNNT---TNNEEIKNCSFNITTELRDKKKKVYALFYKLDVVPIDDNN----SYR

ConS CTNVNVTNTTNNTEEKGEIKNCSFNITTELRDKKKKVYALFYRLDVVPIDDNNNNSSNYR

-- -- -- - ---||---V2-domain------- ---------- --

ConM LINCNTSAITQACPKVSFEPIPIHYCAPAGFAILKCNDKKFNGTGPCKNVSTVQCTHGIK

ConS LINCNTSAITQACPKVSFEPIPIHYCAPAGFAILKCNDKKFNGTGPCKNVSTVQCTHGIK

------------||---------------C2-domain----------------------

ConM PVVSTQLLLNGSLAEEEIIIRSENITNNAKTIIVQLNESVEINCTRPNNNTRKSIRIGPG

ConS PVVSTQLLLNGSLAEEEIIIRSENITNNAKTIIVQLNESVEINCTRPNNNTRKSIRIGPG

-------------------------------------------||-----V3-loop---

ConM QWFYATGDIIGDIRQAHCNISRTKWNKTLQQVAKKLREHFN-KTIIFNPSSGGDLEITTH

ConS QWFYATGDIIGDIRQAHCNISGTKWNKTLQQVVKKLREHFNNKTIIFNPSSGGDLEITTH

----------------||--- ---------- -------- -----C3-domain----

ConM SFNCGGEFFYCNTSELFNSTWNG--------TNNTITLPCRIKQIINMWQRVGQAMYAPP

ConS SFNCGGEFFYCNTSGLFNSTWIGNGTKNNNNTNDTITLPCRIKQIINMWQRVGQPMYAPP

----------||-- ------ - V4 loop||---C4-domain-- -----

ConM IEGKIRCTSNITGLLLTRDGGNNN---TETFRPGGGDMRDNWRSELYKYKVVKIEPLGVA

ConS IQGKIRCVSNITGLLLTRDGGNNNTNETETFRPGGGDMRDNWRSELYKYKVVKIEPLGVA

- ----- -----------||V5lp||-b24--||----C5-domain------------

ConM PTRCKRRVVERRR----RRRAVGIGAVFLGFLGAAGSTMGAASMTLTVQARNLLSGIVQQ

ConS PTRCKRRVVEGGGGSGGGGSAVGIGAVFLGFLGAAGSTMGAASMTLTVQARNLLSGGSGS

------------|| --Heptad-repeat-helices-1-----------

ConM QSNLLRAPECQQHLLQLTVWGIKQLQARVLAVERYLKDQQLLGIWGCSGKLICCTNVPWN

ConS GS-------------GSTVWGIKQLQARVLAVERYLRDQQLLGIWGCSGKLICCTNVPWN

- ------------------- --------||CC-lp||------

ConM SSWSNKSQDEIWDNMTWMEWDKEINNYTDIIYSLIEESQNQQEKNEQELLALD

ConS SSWSNKSQDEIWDNMTWMEWDKEINNYTDIIYSLIEESQNQQEKNEQDLLALD

-------Heptad—Repeat—Helices-2----------------- -----

Table S7 Alignment of the immunogens to the V1V2V3 and V5 sequences of Env

V1 region

ConM CVKLTPLCVTLNCTDVNATNN--TTN---NEEIKNC

ConS CVKLTPLCVTLNCTNVNVTNT--TNNTEEKGEIKNC

Mos3·1 CVKLTPLCVTLNCTNYEGNGNYTTVQNNTIGEIKNC

Mos3·2 CVKLTPLCVTLNCSNVNSNR---TVDNATQGEMKNC

V2 region

ConM SFNITTELRDKKKKVYALFYKLDVVPIDDNN--S--YRLINCNTSAITQAC

ConS SFNITTELRDKKKKVYALFYRLDVVPIDDNNNNSSNYRLINCNTSAITQAC

Mos3·1 SFNITTALRDKVKKVYALFYRLDVVPIKDT-NDSRTYRLINCNTSVITQAC

Mos3·2 SFNITTELRDKKKKVYALFYKLDILPLNGN-NDSNEYRLINCNTSAITQAC

V3 region

ConM TRPNNNTRKSIRIGPGQWFYATGDIIGDIRQAH

ConS TRPNNNTRKSIRIGPGQWFYATGDIIGDIRQAH

Mos3·1 TRPNNNTRKSIRIGPGRWFYATGDIIGDIRRAH

Mos3·2 TRPNNNTRKSIRIGPGQWFYATGEIIGDIRQAH

V5 region

ConM GGNNN---TETF

ConS GGNNNTNETETF

Mos3·1 -GGNHTSETETF

Mos3·2 GGNSENNTKETF

Amino acid differences to ConM highlighted in grey, potential N-linked glycan sites in red (as determined using https://www.hiv.lanl.gov/cgi-bin/GLYCOSITE/glycosite_main.cgi)
