# Supplementary figures and images for: Experimental medicine study with stabilised native-like HIV-1 Env immunogens drives long-term antibody responses, but lacks neutralising breadth
Source: eBioMedicine. 2025 Jan 2;112:105544. doi: 10.1016/j.ebiom.2024.105544 (PMC11753977; doi:10.1016/j.ebiom.2024.105544)

a.

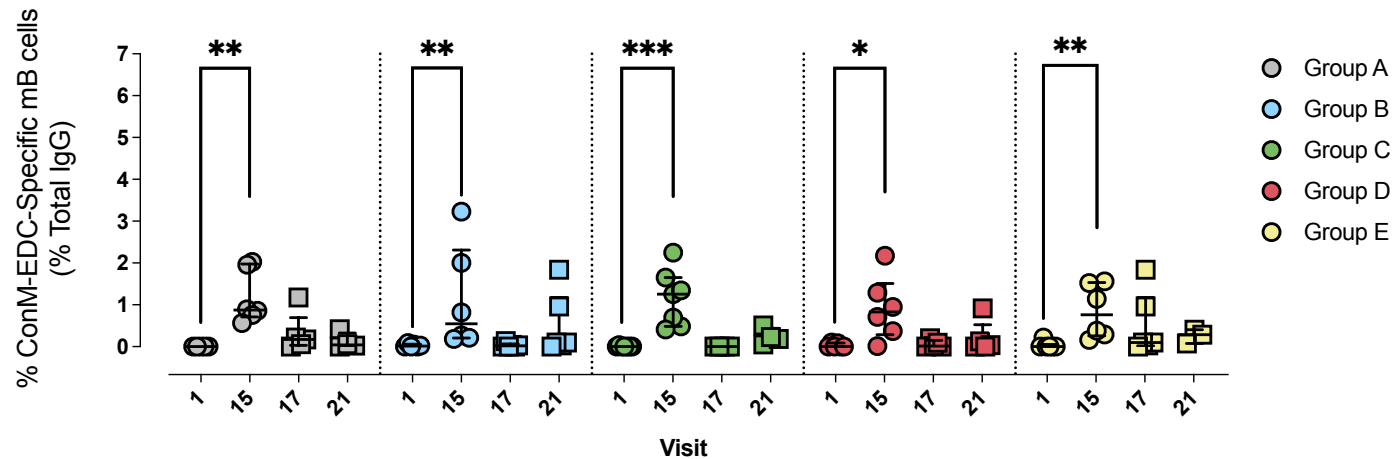

b.

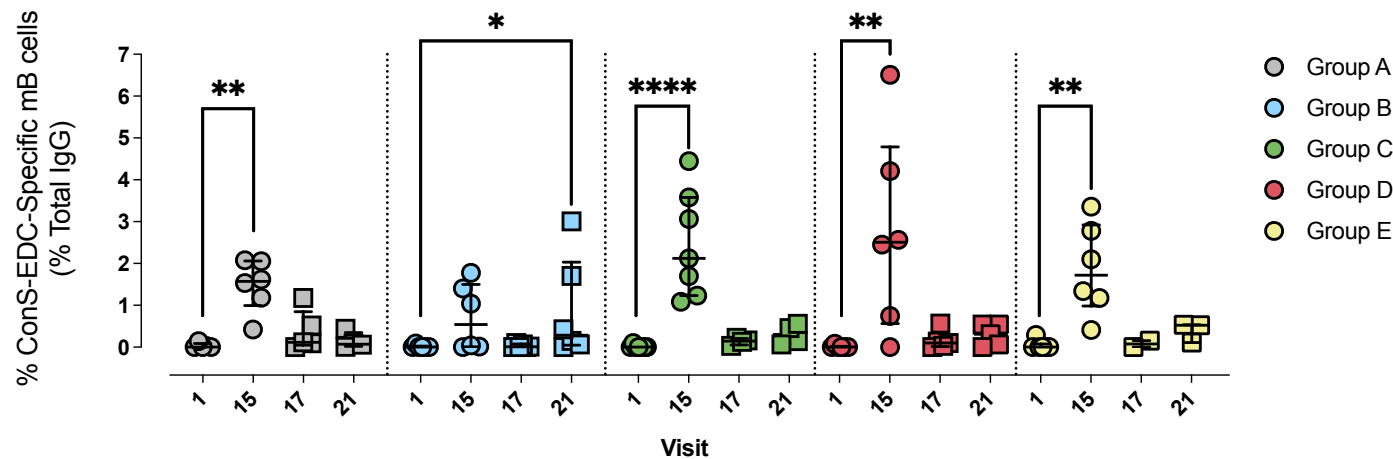

Supplement: Supplementary Fig. S1 — Figure S1 Memory B Cell ELISpot responses to the study-specific proteins in all participants of the EAVI2020_01 Experimental Medicine Study. % antigen-specific memory B cells are shown when PBMC from participants of the study were stimulated with either (a) ConM-EDC or (a) ConS-EDC. The timepoints assessed were V17 (time of Mosaic Boost Injection) and V21 (28 days post-Mosaic Boost). In part 1 (circles), Group A (Grey) received three injections with 100 μg ConM, Group B (Light Blue) received three injections with 100 μg ConM-EDC, Group C (Green) received three injections with 100 μg ConS, Group D (Pink) received three injections with 100 μg ConS-EDC and Group E (Yellow) received two injections with 100 μg ConS followed by one injection with 100 μg ConM. All groups were boosted with a cocktail of 50 μg Mosaic 3.1 and 50 μg Mosaic 3.2 during the fourth injection (part 2 (squares)). All injections were adjuvanted with 500 μg MPLA. All data have been background subtracted. Median values with IQR are shown. Mann-Whitney Test was performed to compare the statistical differences between V17 and V21 within each group, as well as a Kruskal-Wallis Test with Dunn’s multiple comparisons between groups at V17 and V21. [file mmc6.pdf]

a.

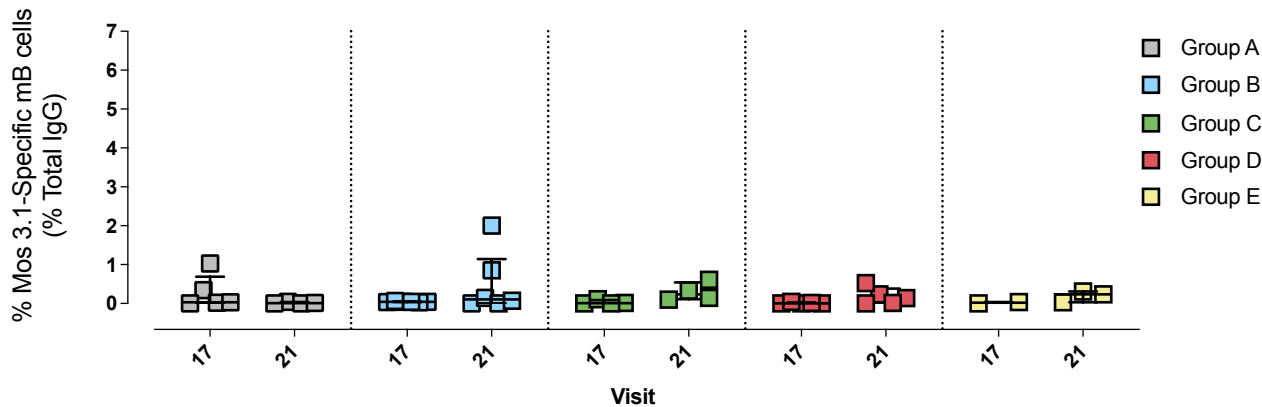

**b.**

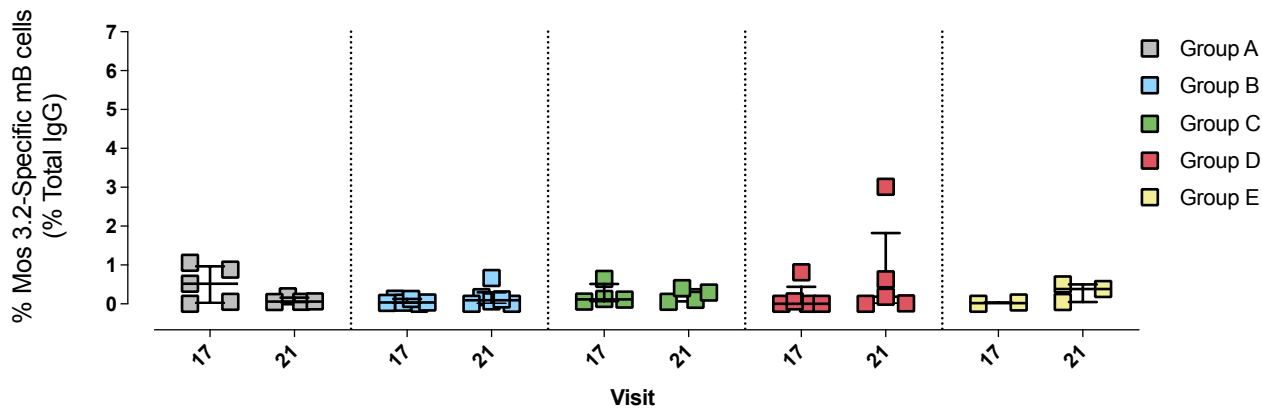

Supplement: Supplementary Fig. S2 — Figure S2 Memory B Cell ELISpot responses to the study-specific proteins in all participants of the EAVI2020_01 Experimental Medicine Study. % antigen-specific memory B cells are shown when PBMC from participants of the study were stimulated with either (a) Mos 3.1 or (b) Mos 3.2. The timepoints assessed were V17 (time of Mosaic Boost Injection) and V21 (28 days post-Mosaic Boost). In part 1, Group A (Grey) received three injections with 100 μg ConM, Group B (Light Blue) received three injections with 100 μg ConM-EDC, Group C (Green) received three injections with 100 μg ConS, Group D (Pink) received three injections with 100 μg ConS-EDC and Group E (Yellow) received two injections with 100 μg ConS followed by one injection with 100 μg ConM. All groups were boosted with a cocktail of 50 μg Mosaic 3.1 and 50 μg Mosaic 3.2 during the fourth injection (part 2 (squares)). All injections were adjuvanted with 500 μg MPLA. All data have been background subtracted. Median values with IQR are shown. Mann-Whitney Test was performed to compare the statistical differences between V17 and V21 within each group, as well as a Kruskal-Wallis Test with Dunn’s multiple comparisons between groups at V17 and V21. [file mmc7.pdf]

a.

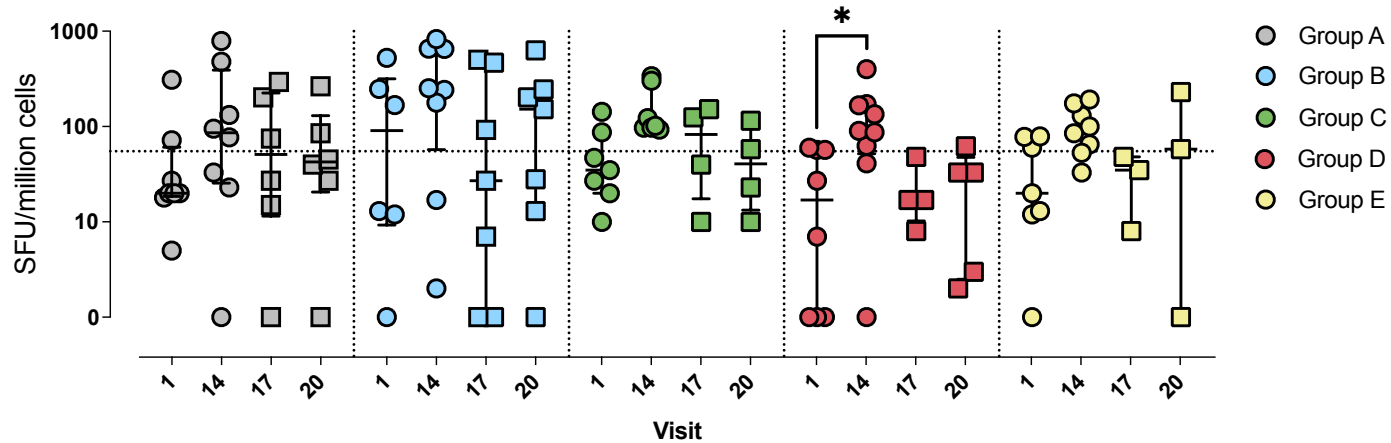

b.

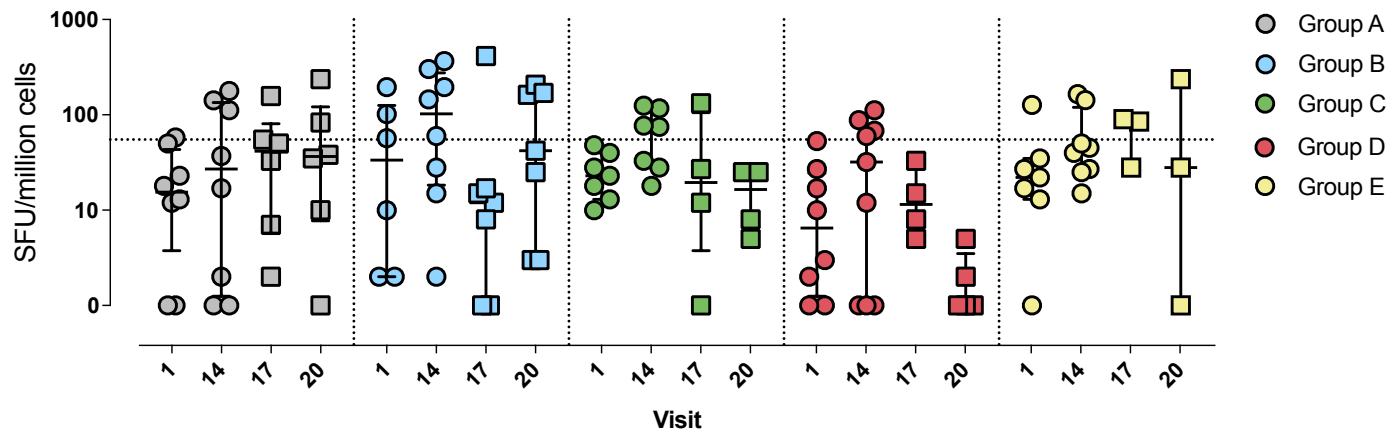

Supplement: Supplementary Fig. S3 — Figure S3 IFN-g ELISpot responses to the study-specific peptide pools in all participants of the EAVI2020_01 experimental medicine study. Spot Forming Units (SFU) per million cells are shown when PBMC participants of the study were stimulated with either (a) Consensus Peptide Pools, or (b) Mosaic Peptide Pools. The timepoints assessed were V1 (time of first injection), V14 (14 days post-third IM injection), V17 (time of Mosaic Boost Injection) and V20 (14 days post-Mosaic Boost). In part 1 (circles), Group A (Grey) received three injections with 100 μg ConM, Group B (Light Blue) received three injections with 100 μg ConM-EDC, Group C (Green) received three injections with 100 μg ConS, Group D (Pink) received three injections with 100 μg ConS-EDC and Group E (Yellow) received two injections with 100 μg ConS followed by one injection with 100 μg ConM. All groups were boosted with a cocktail of 50 μg Mosaic 3.1 and 50 μg Mosaic 3.2 during the fourth injection (part 2 (squares)). All injections were adjuvanted with 500 μg MPLA. All data have been background subtracted and the line at 55 SFU/million cells represents the cutoff for positivity. Median values with IQR are shown. The Kruskal-Wallis with Dunn’s multiple correction test was performed to compare the statistical differences between V1 the remaining timepoints within each group, as well as a comparison between groups at V14, V17 and V20. ∗ <0.05. [file mmc8.pdf]

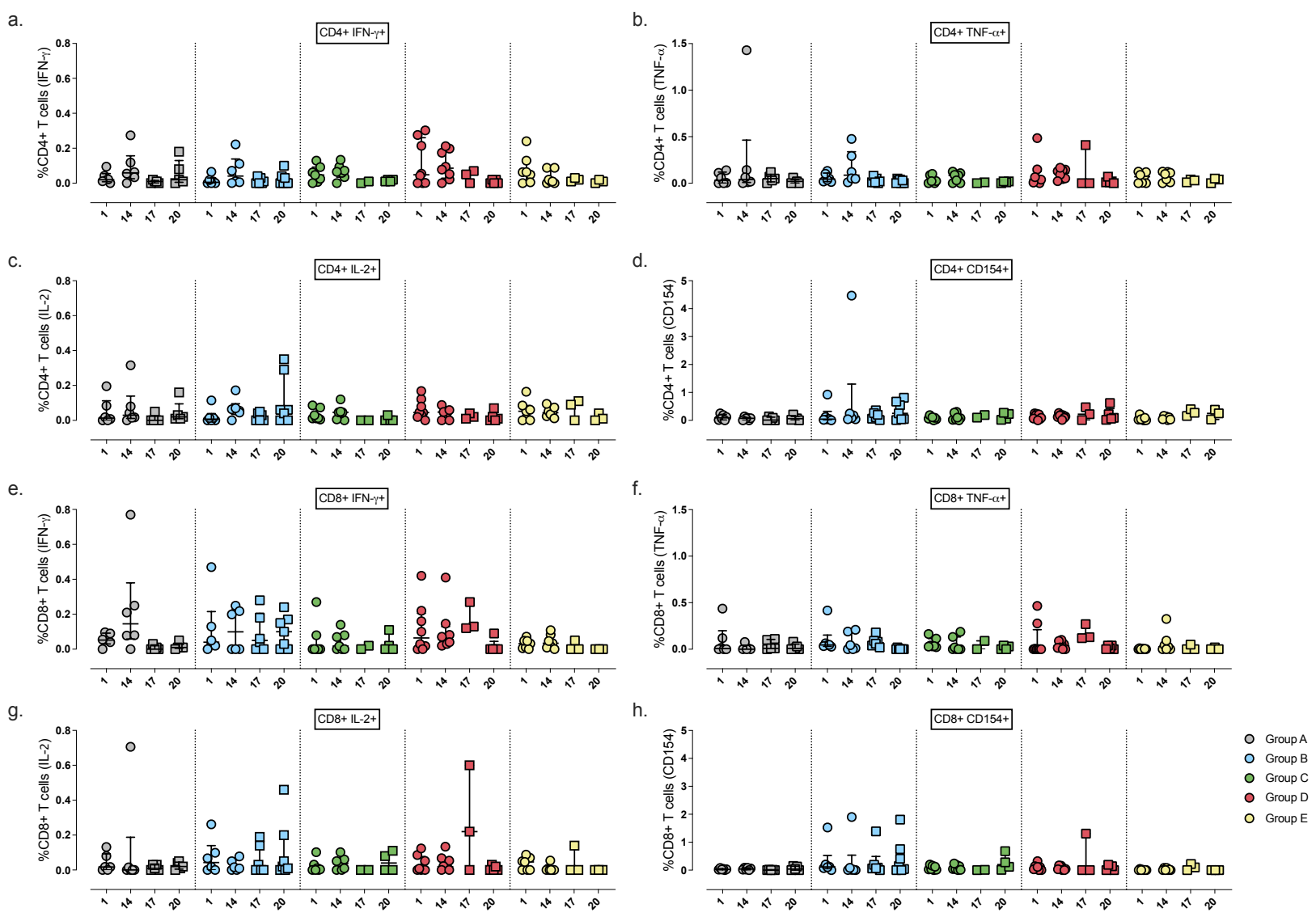

Supplement: Supplementary Fig. S5 — Figure S5 Intracellular Cytokine Staining (ICS) responses to the study-specific Mosaic peptide pools in PBMC from participants of the EAVI2020_01 experimental medicine study. CD4+ IFN- γ (a), TNF-α (b), IL-2 (c), CD154 (d) and CD8+ IFN-γ (e), TNF-α (f), IL-2 (g), CD154 (h) responses are shown as % of live parent. The timepoints assessed were V1 (time of first injection), V14 (14 days post-third IM injection), V17 (time of Mosaic Boost Injection) and V20 (14 days post-Mosaic Boost). In part 1 (circles), Group A (Grey) received three injections with 100 μg ConM, Group B (Light Blue) received three injections with 100 μg ConM-EDC, Group C (Green) received three injections with 100 μg ConS, Group D (Pink) received three injections with 100 μg ConS-EDC and Group E (Yellow) received two injections with 100 μg ConS followed by one injection with 100 μg ConM. All groups were boosted with a cocktail of 50 μg Mosaic 3.1 and 50 μg Mosaic 3.2 during the fourth injection (part 2 (squares)). All injections were adjuvanted with 500 μg MPLA. All data have been background subtracted. Median values with IQR are shown. The Kruskal-Wallis with Dunn’s multiple correction test was performed to compare the statistical differences between V1 the remaining timepoints within each group, as well as a comparison between groups at V14, V17 and V20. [file mmc10.pdf]

a.

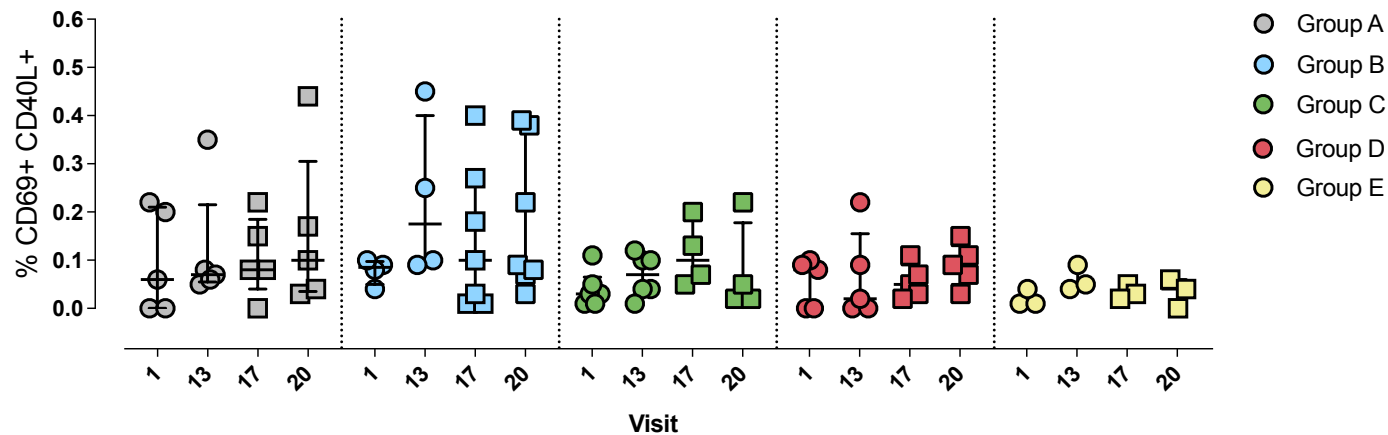

b.

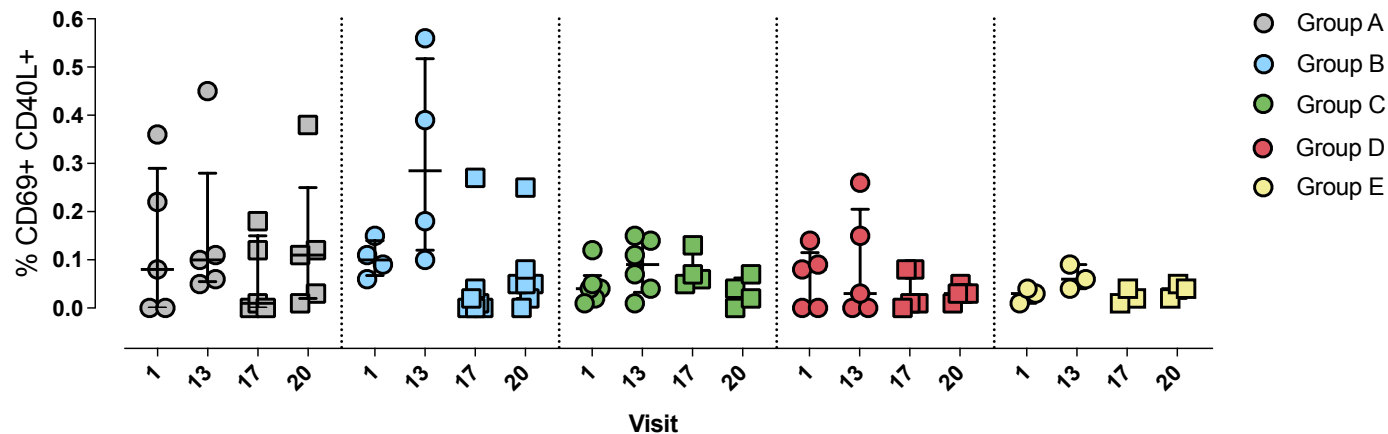

Supplement: Supplementary Fig. S6 — Figure S6 Activation Induced Assay (AIM) responses to the study-specific peptide pools in PBMC from participants of the EAVI2020_01 experimental medicine study. CD4+ CD69+CD40L+ are shown in response to (a) Consensus Peptide Pools 1 and 2 or (b) Mosaic Peptide Pools 1 and 2. The timepoints assessed were V1 (time of first injection), V13 (7 days post-third IM injection), V17 (time of Mosaic Boost Injection) and V20 (14 days post-Mosaic Boost). In part 1 (circles), Group A (Grey) received three injections with 100 μg ConM, Group B (Light Blue) received three injections with 100 μg ConM-EDC, Group C (Green) received three injections with 100 μg ConS, Group D (Pink) received three injections with 100 μg ConS-EDC and Group E (Yellow) received two injections with 100 μg ConS followed by one injection with 100 μg ConM. All groups were boosted with a cocktail of 50 μg Mosaic 3.1 and 50 μg Mosaic 3.2 during the fourth injection (part 2 (squares)). All injections were adjuvanted with 500 μg MPLA. Median values with IQR are shown. The Kruskal-Wallis with Dunn’s multiple correction test was performed to compare the statistical differences between V1 the remaining timepoints within each group, as well as a comparison between groups at V14, V17 and V20. [file mmc11.pdf]

a.

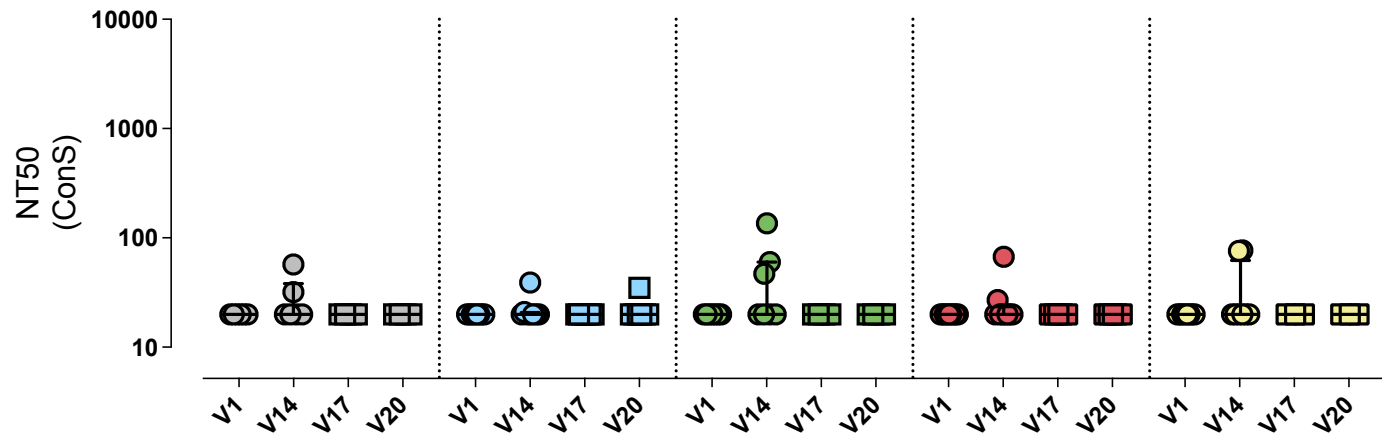

b.

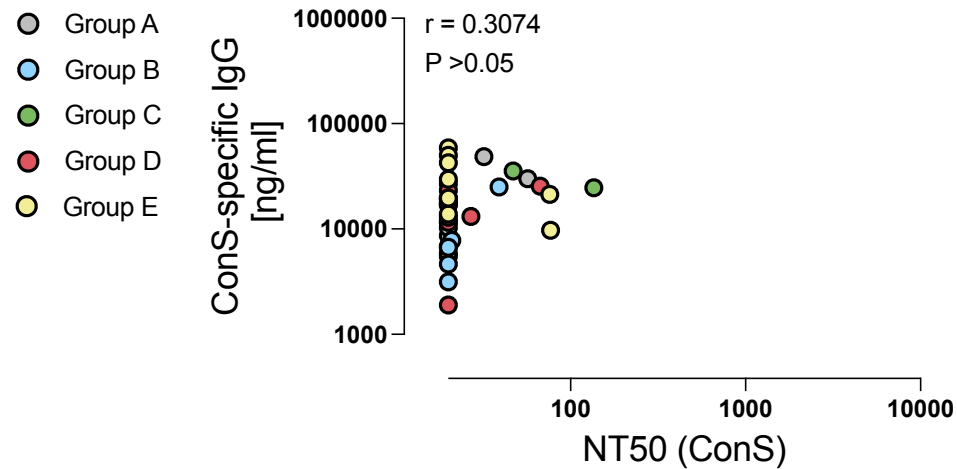

Supplement: Supplementary Fig. S7 — Figure S7 virus neutralisation assay responses in serum samples from participants of the eavi2020_01 experimental medicine study. Serum samples were tested against (a) ConS HIV-1 virus. The timepoints assessed were V1 (time of first infection), V14 (14 days post-third IM injection), V17 (time of Mosaic Boost injection) and V20 (14 days post-Mosaic Boost). These responses were then compared to ConS-specific IgG serum concentrations (b) at V20 (2 weeks post-third injection). In part 1 (circles), Group A (Grey) received three doses of 100 mg ConM, Group B (Light Blue) received three doses of 100 μg ConM-EDC, Group C (Green) received three doses of 100 μg ConS, Group D (Pink) received three doses of 100 μg ConS-EDC and Group E (Yellow) received two doses of 100 μg ConS followed by one doses of 100 μg ConM. All groups were boosted with a cocktail of 50 μg Mosaic 3.1 and 50 μg Mosaic 3.2 during the fourth injection (part 2 (squares)). All injections were adjuvanted with 500 μg MPLA. For (a) median values with IQR are shown. The Kruskal-Wallis with Dunn’s multiple correction test was performed to compare the statistical differences between V1 the remaining timepoints within each group, as well as a comparison between groups at V14, V17 and V20. ∗ <0.05; ∗∗<0.01. For (b), Spearman’s correlation was performed, and the r and p values are shown within the graphs. [file mmc12.pdf]
