# Supplementary material for: Experimental medicine study with stabilised native-like HIV-1 Env immunogens drives long-term antibody responses, but lacks neutralising breadth
Source: eBioMedicine. 2025 Jan 2;112:105544. doi: 10.1016/j.ebiom.2024.105544 (PMC11753977; doi:10.1016/j.ebiom.2024.105544)
Supplement: Supplementary Fig. S4 — Figure S4 Intracellular Cytokine Staining (ICS) responses to the study-specific Consensus peptide pools in PBMC from participants of the EAVI2020_01 experimental medicine study. CD4+ IFN- γ (a), TNF-α (b), IL-2 (c), CD154 (d) and CD8+ IFN-γ (e), TNF-α (f), IL-2 (g), CD154 (h) responses are shown as % of live parent. The timepoints assessed were V1 (time of first injection), V14 (14 days post-third IM injection), V17 (time of Mosaic Boost Injection) and V20 (14 days post-Mosaic Boost). In part 1 (circles), Group A (Grey) received three injections with 100 μg ConM, Group B (Light Blue) received three injections with 100 μg ConM-EDC, Group C (Green) received three injections with 100 μg ConS, Group D (Pink) received three injections with 100 μg ConS-EDC and Group E (Yellow) received two injections with 100 μg ConS followed by one injection with 100 μg ConM. All groups were boosted with a cocktail of 50 μg Mosaic 3.1 and 50 μg Mosaic 3.2 during the fourth injection (part 2 (squares)). All injections were adjuvanted with 500 μg MPLA. All data have been background subtracted. Median values with IQR are shown. The Kruskal-Wallis with Dunn’s multiple correction test was performed to compare the statistical differences between V1 the remaining timepoints within each group, as well as a comparison between groups at V14, V17 and V20. ∗ <0.05. [file mmc9.pdf]

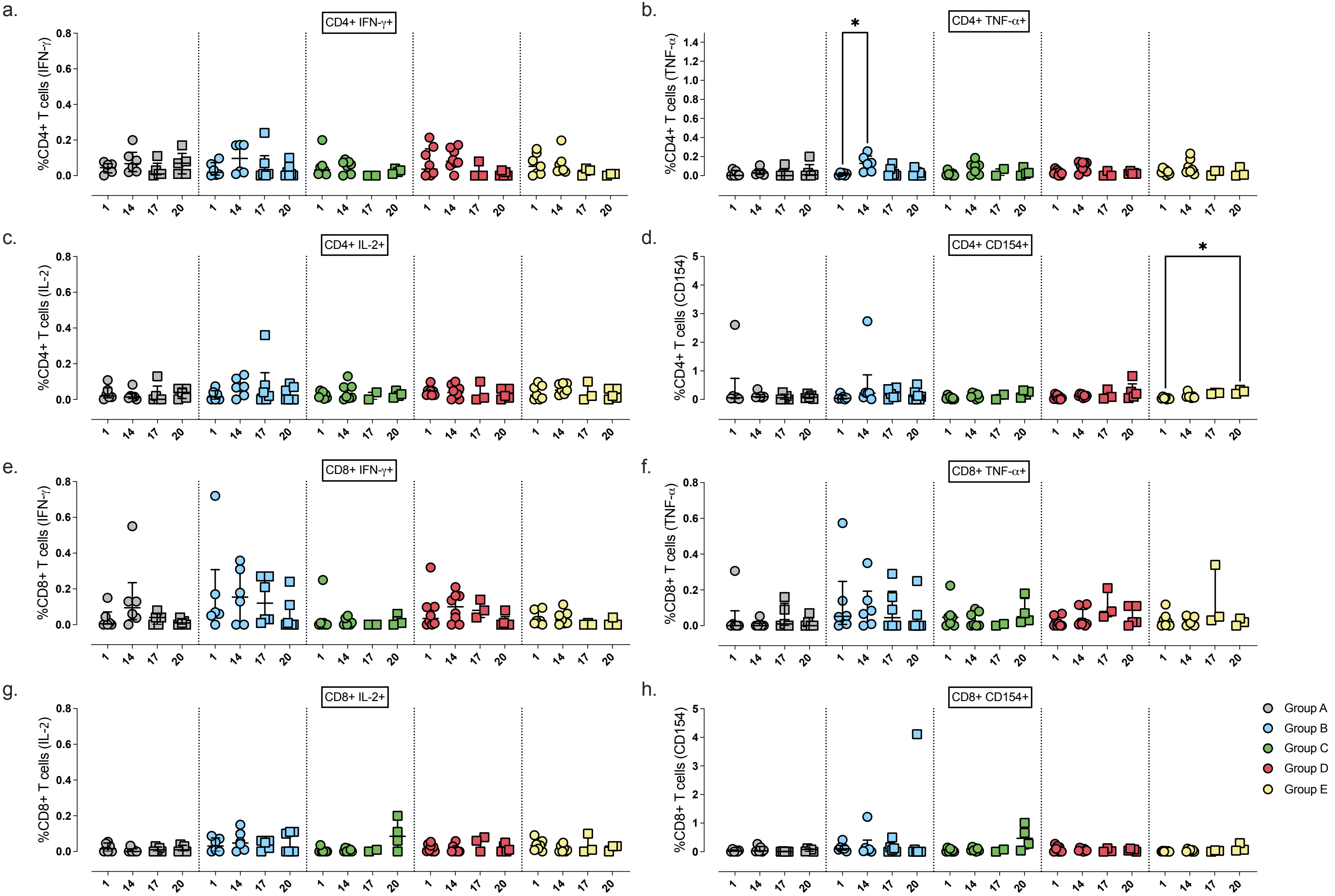
a.
CD4+ IFN-γ+
%CD4+ T cells (IFN-γ)
b.
CD4+ TNF-α+
%CD4+ T cells (TNF-α)
*
c.
CD4+ IL-2+
%CD4+ T cells (IL-2)
d.
CD4+ CD154+
%CD4+ T cells (CD154)
*
e.
CD8+ IFN-γ+
%CD8+ T cells (IFN-γ)
f.
CD8+ TNF-α+
%CD8+ T cells (TNF-α)
g.
CD8+ IL-2+
%CD8+ T cells (IL-2)
h.
CD8+ CD154+
%CD8+ T cells (CD154)
1 14 17 20
Group A
Group B
Group C
Group D
Group E
