# Supplementary material for: Experimental medicine study with stabilised native-like HIV-1 Env immunogens drives long-term antibody responses, but lacks neutralising breadth
Source: eBioMedicine. 2025 Jan 2;112:105544. doi: 10.1016/j.ebiom.2024.105544 (PMC11753977; doi:10.1016/j.ebiom.2024.105544)
Supplement: Supplementary file 7 [file mmc3.docx]

REAGENT or RESOURCE	SOURCE	IDENTIFIER
**Antibodies**		
anti-human CD3 BV650 (Clone OKT3), flow cytometry	BioLegend	Cat #: 317324; RRID:AB_2563352
anti-human CD4 PerCP-Cy5·5 (RPA-T4), flow cytometry	BioLegend	Cat #: 300530; RRID:AB_893322
anti-human CD8 AF700 (RPA-T8), flow cytometry	BioLegend	Cat #: 301028; RRID:AB_493745
anti-human IFN-γ AF488 (4S·B3), flow cytometry	BioLegend	Cat #: 502515; RRID:AB_493029
anti-human TNF-α PE-Cy7 (MAb11), flow cytometry	BioLegend	Cat #: 502930; RRID:AB_2204079
anti-human IL-2 BV510 (MQ1-17H12), flow cytometry	BioLegend	Cat #: 500338; RRID:AB_2562632
anti-human CD154 BV421 (24-31), flow cytometry	BioLegend	Cat #: 310824; RRID:AB_2562721
anti-human CD40 blocking antibody, flow cytometry	Miltenyi Biotech	Cat #: 130-094-133; RRID:AB_10839704
anti-human CXCR5-BB515 (Clone RF8B2), flow cytometry	BD Biosciences	Cat #: 564624; RRID:AB_2738871
anti-human CD3 BUV395 (UCHT1), flow cytometry	BD Biosciences	Cat #: 563546; RRID:AB_2744387
anti-human CD4 BUV496 (SK3), flow cytometry	BD Biosciences	Cat #: 612936; RRID:AB_2870220
anti-human CD8 V500 (RPA-T8), flow cytometry	BD Biosciences	Cat #: 560774; RRID:AB_1937325
anti-human CD14 V500 (M5E2), flow cytometry	BD Biosciences	Cat #: 561391; RRID:AB_10611856
anti-human CD19 V500 (H1B19), flow cytometry	BD Biosciences	Cat #: 561121; RRID:AB_10562391
anti-human CD154 PE (TRAP-1), flow cytometry	BD Biosciences	Cat #: 555700; RRID:AB_396050
anti-human CD45RA PE-Dazzle (HI100), flow cytometry	BioLegend	Cat #: 304146; RRID:AB_2564079
anti-human PD-L1 PE-Cy7 (29E.2AE), flow cytometry	BioLegend	Cat #: 329718; RRID:AB_2561687
anti-human OX40 APC (ACT35), flow cytometry	BioLegend	Cat #: 350008; RRID:AB_10719958
anti-human CD25 APC-Fire750 (BC96), flow cytometry	BioLegend	Cat #: 302642; RRID:AB_2632839
anti-human CD69 BV650 (FN50), flow cytometry	BioLegend	Cat #: 310934; RRID:AB_2563158
anti-human PD-1 BV421 (EH12·2H7), flow cytometry	BioLegend	Cat #: 329920; RRID:AB_10960742
anti-human CD3, IFN-γ ELISpot	Mabtech	Cat #: 3605-1-1000; RRID:AB_907218
Coating Antibody/Positive control: Anti-human IgG mAb MT91/145, IFN-γ ELISpot	Mabtech	Cat #: 3850-3-250/1000; RRID:AB_10665549
Detection Antibody: Anti-human IgG, IFN-γ ELISpot	Mabtech	Cat #: 3850-6-250/1000;RRID:AB_10666158
**Virus Strains**		
ConS gp160 pcDNA3.1 #564 AmpR	Robin Shattock (Imperial College London)	N/A
LAI conM	Bontjer MC and Sanders, R.W. (Amsterdam UMC)	N/A
**Biological Samples**		
Blood samples from healthy participants	NIHR Imperial CRF, Hammersmith Hospital	N/A
**Chemicals, Peptides or Recombinant Proteins**		
Human serum	Sigma	Cat #: H4522
Heat-Inactivated Foetal Bovine Serum, USA Origin	Sigma	Cat #: F4135
Penicillin-Streptomycin	Gibco	Cat #: 15140-122
L-glutamine	Gibco	Cat #: 25030-024
Cell Stimulation Cocktail	Invitrogen	Cat #: 00-4970-93
CEFX Super stim peptide pool	JPT	Cat #: PM-CEFX-1
Bovine Serum Albumin	Sigma	Cat #: A7906-100G
DPBS	Gibco	Cat #: 14190-144
PBS	VWR	Cat #: 437117K
ConM SOSIP.v7	PolymunScientificGmbH	Lot #: MSIP528_250919A
ConSOSL.UFO.644	PolymunScientificGmbH	Lot #: T143/SUFO664/B1
Mmos3.1 SOSIP.v7	PolymunScientificGmbH	Lot #: T158/MOS1SIP/B1
Mmos3.2 SOSIP.v7	PolymunScientificGmbH	Lot #: T160/MOS2SIP/B1
Mmos3.3 SOSIPv8.2 S166R	PolymunScientificGmbH	Lot #: M3SIP8_111219A
Consensus Peptide Pool	Genscript	N/A
Mosaic Peptide Pool	Genscript	N/A
AEC solution	BD Biosciences	Cat #: 551951
ELISpot Plus kit. Pre-coated one step detection kit for detecting Human IFN-γ release	Mabtech	Cat #: 3420-4APT-10
Detection Reagent: Streptavidin-HRP	Mabtech	Cat #: 3310-9
TMB substrateSure Blue TMB 1-Component Peroxidase Substrate	KPL	Cat #: 5120-0074 (52-00-02)
TMB stop solution	KPL	Cat #: 5150-0021 (50-85-06)
RPMI-1640 Medium, with 25 mM HEPES	Sigma	Cat #: R5886
Lyophilised recombinant human IL-2	Roche	Cat #: 11 011 456 001
R848	Invitrogen	Cat #: Vac-r848
**Experimental Models: Cell Lines**		
HEK293T	ATCC	Cat #: CRL-3216; RRID:CVCL_0063
TZM-bl	BEI Resources	Cat #: ARP-8129; RRID:CVCL_B478
**Critical Commercial Assays**		
fixable viability dye eFluor780	eBioscience	Cat #: 65-0865-14
fixable viability dye eFluor506	eBioscience	Cat #: 65-0866-14
intracellular (IC) fixation buffer	eBioscience	Cat #: 88-8824-00
Permeabilisation buffer	eBioscience	Cat #: 00-8333-56
Fixation buffer	eBioscience	Cat #: 00-8222-49
Luciferase Assay System	Promega	Cat #: E2620
DEAE-Dextran hydrochloride	Merck	Cat #: D9885-10G
DMEM HIGH GLUCOSE WITH STABLE L-GLUTAMINE	Euroclone	Cat #: ECM0103L
MultiScreen IP 96-well filter plate	Millepore	Cat #: MSIPS4510
